# Brain-targeting drug delivery systems: The state of the art in treatment of glioblastoma

**DOI:** 10.1016/j.mtbio.2025.101443

**Published:** 2025-01-03

**Authors:** Bo Sun, Rong Li, Ning Ji, Han Liu, Hongxiang Wang, Chao Chen, Long Bai, Jiacan Su, Juxiang Chen

**Affiliations:** a Department of Neurosurgery, Shanghai Changhai Hospital, Naval Medical University, Shanghai, 200433, China; b Department of Orthopedics, Xinhua Hospital, Shanghai Jiao Tong University School of Medicine, Shanghai, 200092, China; c Trauma Orthopedics Center, Xinhua Hospital, Shanghai Jiao Tong University School of Medicine, Shanghai 200092, China; d Institute of Musculoskeletal Injury and Translational Medicine of Organoids, Xinhua Hospital, Shanghai Jiao Tong University School of Medicine, Shanghai, 200092, China; e Institute of Translational Medicine, Shanghai University, Shanghai, 200444, China; f National Center for Translational Medicine SHU Branch, Shanghai University, Shanghai, 200444, China

**Keywords:** Glioblastoma, Blood brain barrier, Blood-tumor barrier, Brain targeting drug delivery system, Nanoparticles

## Abstract

Glioblastoma (GBM) is the most prevalent primary malignant brain tumor, characterized by a high mortality rate and a poor prognosis. The blood-brain barrier (BBB) and the blood-tumor barrier (BTB) present significant obstacles to the efficacy of tumor-targeted pharmacotherapy, thereby impeding the therapeutic potential of numerous candidate drugs. Targeting delivery of adequate doses of drug across the BBB to treat GBM has become a prominent research area in recent years. This emphasis has driven the exploration and evaluation of diverse technologies for GBM pharmacotherapy, with some already undergoing clinical trials. This review provides a thorough overview of recent advancements and challenges in targeted drug delivery for GBM treatment. It specifically emphasizes systemic drug administration strategies to assess their potential and limitations in GBM treatment. Furthermore, this review highlights promising future research directions in the development of intelligent drug delivery systems aimed at overcoming current challenges and enhancing therapeutic efficacy against GBM. These advancements not only support foundational research on targeted drug delivery systems for GBM but also offer methodological approaches for future clinical applications.

## Highlights

• Comprehensive strategies and applications of non-invasive systemic drug delivery for GBM treatment are discussed.
• Detailed exploration of liposomes and cell membrane-coated nanoparticles is presented.
• Mammal, bacterial, and plant-derived extracellular vesicles show promise in therapeutic transport.
• The role of viral vectors, particularly adeno-associated viruses, in GBM gene therapy is detailed.
• Key development objectives and strategies for clinical translation of GBM-targeted drug delivery systems are highlighted.

## Graphical abstract

**Figure:**
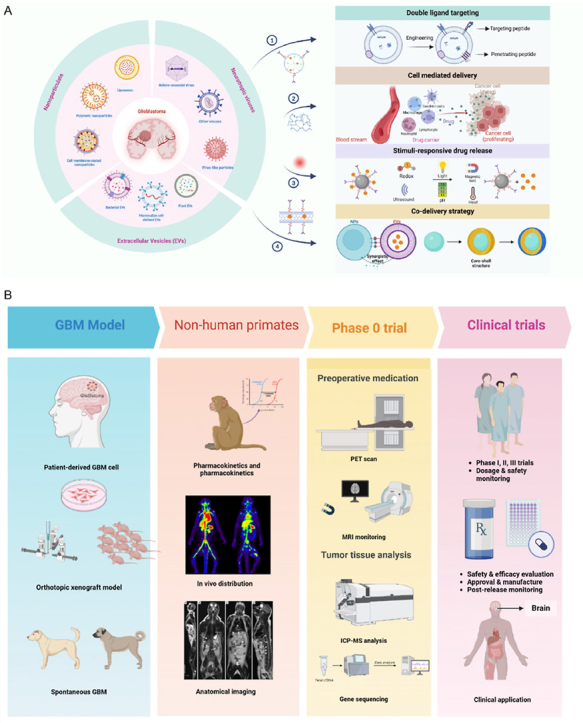

## 1 Introduction

Glioblastoma (GBM) is a prevalent primary intracranial malignant tumor that originates from glial cells, characterized by its short median survival and a five-year survival rate of lower than 5 % [1]. The most common approach involves surgical tumor removal, yet achieving total resection is challenging due to the tumor's diffuse infiltrative growth, strong invasiveness, and rapid vascular proliferation. Although postoperative adjuvant chemoradiotherapy can enhance the therapeutic outcomes, the recurrence of residual tumor tissues often leads to a poor prognosis for patients. Hence, finding effective therapeutic strategies for promptly addressing GBM is imperative.

Despite the development of numerous tumor treatment drugs in recent years, the blood-brain barrier (BBB) and blood-tumor barrier (BTB) often limit the penetration of therapeutic agents into intracranial tumors. So far, only five drugs have been approved by the US Food and Drug (FDA) for the treatment of GBM. Among them, excluding bevacizumab, the small-molecule drugs lomustine, carmustine, temozolomide (TMZ), and vorasidenib are capable of crossing the BBB [2,3]. However, their sensitivity against GBM cells is significantly lower compared to other common anti-cancer agents like paclitaxel (PTX) [4]. Therefore, overcoming the BBB and the BTB remains crucial for effective brain drug delivery. The advancement of nanomedicine has enabled the potential for precise pharmacotherapeutic targeting in GBM treatment, thereby providing more options for the drug therapy of this highly aggressive brain tumor. These drug delivery systems can penetrate the BBB and reduce accumulation in peripheral organs [5]. Moreover, they can selectively release drugs within the specific tumor microenvironment [6]. Consequently, brain-targeted drug delivery systems hold great potential in the treatment of GBM.

This review highlights recent advancements in brain delivery technologies for GBM therapy, with a focus on biopharmaceutical delivery. Invasive drug delivery methods such as intracranial injections and convection-enhanced delivery (CED) pose significant risks of brain damage, including edema, inflammation, and infection, necessitating specialized surgical procedures [7]. Herein, this review emphasizes non-invasive systemic drug administration technologies. Currently, various nanomaterials are under investigation in preclinical studies for GBM, encompassing liposomes, micelles, dendrimers, inorganic nanoparticles, hybrid nanoparticles, and exosomes, among others. Based on their characteristics, brain-targeted drug delivery systems are classified into three main categories: nanoparticulate drug delivery systems, extracellular vesicles, and neurotropic viruses. We systematically summarize the development of different nanoparticulate drug delivery systems and introduce the advantages and disadvantages of extracellular vesicles from different sources (mammals, bacteria and plants) in drug delivery for GBM treatment. Alongside the advancement of gene therapy, the use of viruses as vectors for delivering gene therapy agents has emerged as a significant and critical consideration in the field. Therefore, we also introduce the applications of neurotropic viruses in the treatment of GBM in recent years. Finally, future pathways for the advancement of drug delivery systems in the treatment of GBM are also proposed.

## 2 BBB alteration and targeting delivery strategies

### 2.1 Blood–brain barrier and blood-tumor barrier

**Fig. 1:**
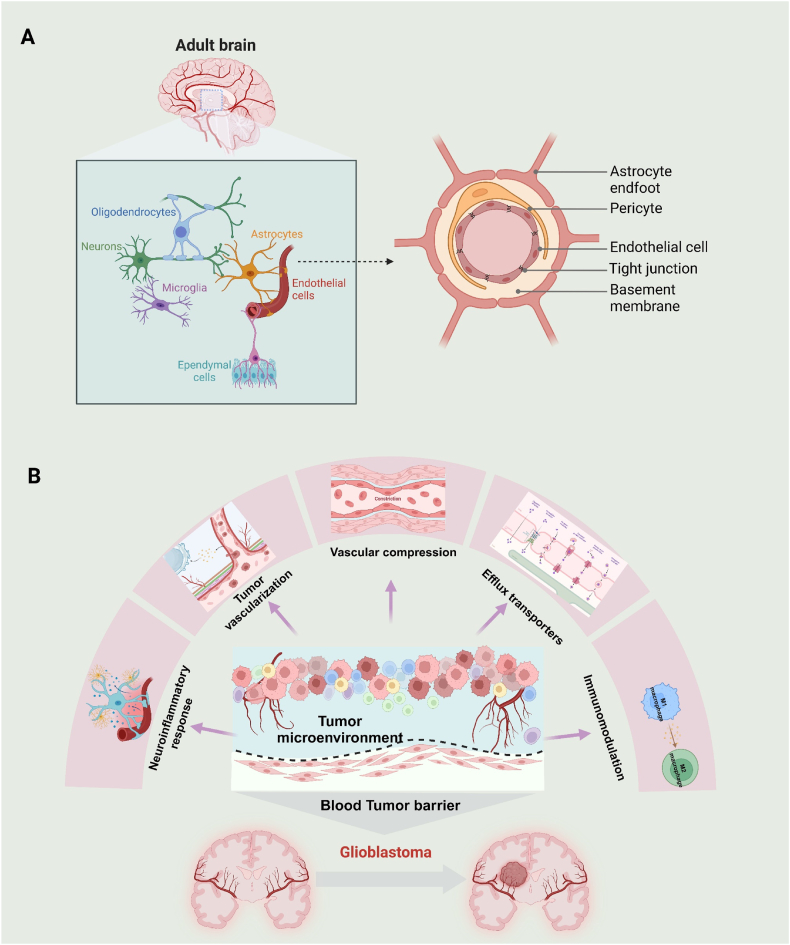
Overview of the BBB and BTB. (A) Schematic illustration of BBB structure. The endothelial cells form a monolayer lining the capillaries within the brain, tightly connected by tight junction protein. This layer is surrounded by a continuous basal lamina, which includes components such as laminin, collagen type IV, fibronectin, heparan sulfate glycoprotein, and other matrix proteins [11]. The basal lamina acts as a dense, low-permeability filler that reinforces the barrier properties of the BBB. Astrocyte end-feet and pericytes further support this structure, while neuronal synapses and microglia innervate the NVU, facilitating dynamic cellular interactions crucial for BBB maintenance. (B) Alterations as follows in the GBM tumor microenvironment that may impede drug delivery or diminish therapeutic efficacy: (i) The rapid expansion of tumors compresses blood vessels, leading to abnormal blood circulation within the tumor; (ii) The formation of new abnormal blood vessels may increase the heterogeneity of the BTB; (iii) The tumor is surrounded by a neuroinflammatory response, which is accompanied by the migration of activated microglia, astrocytes, and other immune cells to the tumor, forming an encircling structure that resembles a protective barrier; (iv) In the tumor microenvironment, abundant microglia and macrophages predominantly adopt a tumor-promoting M2 phenotype, affecting treatment efficacy; (v) Efflux transporters on the GBM cell membrane can expel antitumor agents from the tumor cells.

The BBB is a critical structure that maintains the brain's internal environment, ensuring homeostasis within the central nervous system. It functions as a neurovascular unit (NUV) composed of several key elements by endothelial cells, pericytes, basal lamina, astrocyte end-feet, and immune cells (Fig. 1A) [8]. The primary function of the BBB is to regulate the transport of essential nutrients, including glucose, amino acids, vitamins, and iron ions, from the blood into the central nervous system (CNS) extracellular fluids [9]. Simultaneously, it restricts the entry of potentially harmful substances, including neurotoxic agents. To achieve this, the BBB employs a range of efflux proteins that selectively expel specific molecules from the brain, adding another layer of selective permeability [8]. Importantly, the BBB presents a significant challenge to brain drug delivery. It restricts the entry of macromolecular drugs and approximately 98 % of small molecule drugs, limiting their therapeutic potential against conditions such as GBM [10].

The development of GBM leads to significant changes in BBB environment, ultimately resulting in the formation of another pathological barrier referred to as BTB (Fig. 1B). Specifically, the BTB consists of specialized endothelial cells surrounding the tumor, exhibiting non-uniform permeability [12]. As tumor growth progresses, it compresses blood vessels, causing increased leakage in the central tumor area in contrast to the surrounding peritumoral region, thus contributing to the high heterogeneity of the BTB. Within the tumor core, the activation of the hypoxia-inducible factor-1a (HIF-1a) signaling pathway stimulates the expression of vascular endothelial growth factor (VEGF), thereby promoting angiogenesis [13]. This mechanism facilitates the formation of new, abnormal blood vessels, resulting in heightened permeability and heterogeneity of the BTB. Meanwhile, the tumor is surrounded by a neuroinflammatory response, commonly referred to as astrogliosis or glial scarring [12]. This neuroinflammatory response creates a complex microenvironment that further complicates drug delivery. In addition, the microenvironment of GBM is characterized by the presence of tumor-promoting M2 phenotype immune cells that facilitate tumor progression and hinder effective treatment [12]. Importantly, many studies show that progressive GBM maintains an intact BBB with efflux transporters that impede the penetration of antitumor agents into the cells [12]. All in all, most therapeutics fail to reach the infiltrating GBM cells, underscoring the limitations of conventional drug for GBM treatment [14].

Given the distinctive microenvironment of the brain, understanding the structural and functional diversity of the BBB and BTB is crucial. GBM induces multifaceted changes in the BTB. Therefore, identifying the critical changes that have functional implications for tumor progression and drug penetration is crucial for designing safe and effective drug delivery systems.

### 2.2 Biological mechanisms of brain delivery

Traditional strategies for drug delivery to the brain focus on improving the permeability of the BBB and optimize the physicochemical properties of therapeutic agents, thereby increasing drug concentrations in the brain [15]. Certain agents, such as hypertonic solutions or vasoactive compounds like bradykinin, can transiently augment BBB permeability, thus boosting the efficacy of co-administered anticancer drugs [16]. Focused ultrasound can also induce transient BBB opening, a technique widely utilized for delivering conventional therapeutics in the treatment of GBM [17]. However, the transient nature of the effects of osmotic agents or ultrasound on BBB permeability, coupled with the predominantly animal-level experimentation, raise concerns about the potential for off-target toxicity and harm to human brain tissue.

In recent years, the transcytotic mechanisms for traversing the BBB have been extensively investigated and proven to be one of the most sophisticated methods for delivering therapeutics to the brain. Nanocarriers incorporating targeting ligands, including proteins, antibodies, and peptides, can adhere to the cell membrane on the brain vascular endothelial cells. This interaction triggers an endocytosis reaction, thereby facilitating the release of therapeutics into the brain [11]. Peptides are commonly used as ligands in research due to their advantages, including ease of synthesis, modification, low immunogenicity, and cost-effective [18]. As a result, various peptides such as Angiopep-2, iRGD have been extensively employed in modifying delivery carriers to enhance drug accumulation in GBM lesions [19,20]. The surface of the nanomaterials is modified with peptides through chemical modification or genetic engineering. This modification allows the ligand to bind specifically to its receptor, facilitating the active delivery of therapeutics across the BBB. Ideally, the target receptor should exhibit high expression on the surface of brain microvascular beds and GBM cells, while being minimally in peripheral vasculature to reduce off-target toxic effects. Designing appropriate drug delivery systems with specific targeting ligands tailored to the characteristics of the BBB in GBM is crucial for clinical translation.

The pursuit of developing efficient drug delivery systems capable of penetrating the BBB and targeting GBM lesions with precision has emerged as a prominent research priority. Notable strategies include nanoparticulate drug delivery systems, extracellular vesicles, and neurotropic viruses, all of which have demonstrated potential for improving therapeutic efficacy and specificity.

## 3 Nanoparticulate drug delivery systems

The application of nanoparticle drug delivery system (NDDS) in the CNS encompasses a range of nanocarriers, such as polymer nanoparticles and liposomes. These NDDS typically chemically synthesized with diameters ranging from 10 to 200 nm and exhibit variations in parameters like chemical compositions and surface properties [11]. NDDS can be tailored in terms of size and shape, with polymeric nanoparticles offering key advantages including facile synthesis, sustained and controlled drug release, and non-immunogenicity [21]. Additionally, to improve the biocompatibility and targeting capabilities of NDDS, various biomimetic nanomaterials were further designed by wrapping the cell membrane.

### 3.1 Polymeric nanoparticles

Polymeric nanoparticles (NPs) for drug delivery encompass a diverse array of nanocarriers, typically synthesized using biocompatible, biodegradable, and poorly soluble polymers. These NPs are often coated with poly (ethylene glycol) (PEG) to enhance their circulation duration within the bloodstream [22]. They offer advantages such as easy synthesis, a large surface area, and stable pharmacokinetics, making them particularly valuable in enhancing the bioavailability of therapeutic agents [23]. Their nano-size and ability to be surface-modified with various molecules, such as ligands, facilitate their crossing of the BBB and targeted delivery to GBM. A variety of synthetic polymers is employed in the delivery of drugs for GBM, including polycaprolactone (PCL), poly(amido)amine (PAMAM), alginates, chitosan, and others [[23], [24], [25]]. Moreover, poly (lactic-co-glycolic acid) (PLGA) NPs have been approved by the U.S. FDA approval due to their excellent properties [26].

**Fig. 2:**
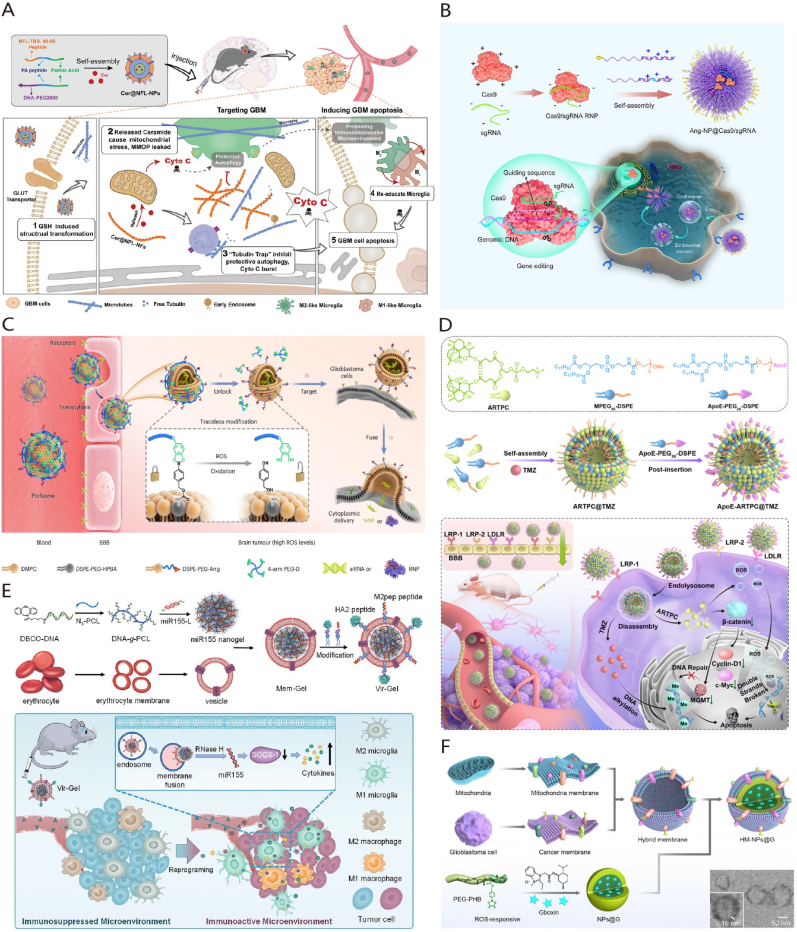
Strategies of nanoparticulate drug delivery systems in the treatment of GBM. (A) Illustration of transformable nanoparticles with biomimetic neurofilament function for interfacing with mitochondrial autophagy in GBM treatment. Adapted with permission from Ref. [28]. Copyright 2022.Wiley-VCH GmbH. (B) Schematic illustration of a functionalized polymeric nanoparticle loaded with Cas9/gRNA RNP complex for the treatment of GBM. Adapted with permission from Ref. [32]. Copyright 2022. Elsevier B.V. (C) Illustration of the use of fusogenic liposome for the targeted delivery of siRNA and Cas9/gRNA RNP complex in the treatment of GBM. Adapted with permission from Ref. [48]. Copyright 2024. Spring Nature. (D) A schematic illustration demonstrating the targeted delivery of dual therapeutic agents using liposomes to treat TMZ-resistant GBM cells. Adapted with permission from Ref. [50]. Copyright 2022. Elsevier B.V. (E) The schematic diagram illustrates the use of RBC membrane-coated nucleic acid nanogel to reprogram microglia and macrophages for the treatment of GBM. Adapted with permission from Ref. [51]. Copyright 2021.Wiley-VCH GmbH. (F) The schematic diagram illustrates the utilization of GBM cell-mitochondria hybrid membrane-coated nanoparticles for the treatment of GBM. Adapted with permission from Ref. [52]. Copyright 2023. Spring Nature.

Utilizing NPs presents a promising strategy for delivering chemotherapeutic agents in GBM treatment. TMZ represents the frontline chemotherapy for treating GBM, but resistance often arises in advanced stages. Zhang et al. [27] devised LAT1-targeting NPs co-loaded with TMZ and sorafenib, which effectively localize to the GBM site, enhancing therapeutic effects through sorafenib-induced ferroptosis. Mitophagy of mitochondria plays a pivotal role in GBM treatment resistance, while neurofilaments can inhibit mitophagy by regulating tubulin polymerization. Researchers developed a supramolecular sequence platform resembling a "neurofilament-like" short fiber to prevent protective autophagy and tumor-associated microenvironment formation (Fig. 2A) [28]. STING agonists have demonstrated potent anti-GBM effects intratumorally, albeit hampered by systemic toxicity and poor pharmacokinetic properties for intravenous administration. Wang et al. [29] engineered peptide-conjugated H-ferritin(HFn) nanoparticles to deliver a STING agonist to GBM, triggering a potent anti-GBM immune response.

In the realm of gene therapy, NPs exhibit unique benefits. While siRNAs typically form nanoparticle solutions by binding to oppositely charged molecules, the abundance of charged biomolecules in the bloodstream decreases their blood circulation time. Zheng et al. [30] devised an Angiopep-2-modified siRNA nanodrug stabilized by triple interactions (electrostatic, hydrogen bonding, and hydrophobic forces), enhancing siRNA stability and BBB penetration. By co-delivering siPLK1 and siEGFR-2 and leveraging reactive oxygen species for siRNA release, target gene expression was downregulated. In addition, CRISPR/Cas9 technology is a prominent GBM treatment method. Researchers engineered polymeric nanocapsules adorned with Angiopep-2 peptide to encapsulate and circulate Cas9/sgRNA ribonucleoprotein (RNP) efficiently, ensuring blood stability and protein activity (Fig. 2B) [31,32]. These studies have demonstrated that NPs can effectively deliver gene therapeutics across the BBB, offering a potent and safe strategy for GBM gene therapy.

NPs are also extensively used in the immunotherapy of GBM. The BBB impedes the penetration of immune checkpoint blockade (ICB) antibodies into the brain, thereby reducing their effectiveness against GBM. Wang et al. [33] developed BBB-penetrating copolymers through free-radical polymerization and subsequently linked them with anti-programmed death ligand 1 (anti-PD-L1). These pH-responsive nanoparticles exhibit substantial BBB penetration capability, enabling the release of anti-PD-L1 within the acidic GBM microenvironment, thereby enhancing the efficacy of ICB therapy. Xie et al. [34] proposed Hsp70-targeting gold nanoparticles with active targeting to enhance doxorubicin (DOX) enrichment in GBM, in combination with anti-PD-1 for chemo-immunotherapy.

Addressing the tumor microenvironment is another critical aspect of GBM therapy. The upregulated CXCL12/CXCR4 signaling pathway in GBM contributes to an immunosuppressive tumor immune microenvironment (TIME) and tumor progression, with AMD3100 as a CXCR4 inhibitor. However, due to its poor pharmacokinetics and unfavorable bioavailability, the clinical utility of AMD3100 is limited. Mahmoud et al. [35] developed synthetic protein nanoparticles (SPNPs) loaded with AMD3100 to target the CXCL2/CXCR4 pathway in GBM. The study showed that CXCR4 blocking led to reduced infiltration of monocyte-derived suppressor cells within the TIME in vivo, enhancing the sensitivity of GBM cells to radiation-induced immunogenic cell death (ICD) and triggering anti-GBM-specific immunological T-cell-mediated immunity.

Over the past few years, there has been a substantial increase in research on polymeric NPs for GBM treatment. The expanding variety of organic compounds has resulted in the identification of numerous new polymers that can be employed for precise drug delivery to the brain. Furthermore, natural polymers such as gelatin, albumin, and lactoferrin nanoparticles are favored for drug delivery due to their non-toxic, non-immunogenic, and BBB-permeable characteristics. Representative NPs in GBM treatment are summarized in Table 1.

**Table 1:** Summary of representative NPs and liposomes in GBM treatment.

Nanoparticles	Ligand	Targeted site	Average Size	Therapeutic agents	Cell line	Ref.
PEG-P (TMC-DTC)	ApoE peptide	LDLR	42–45 nm	GrB; CpG	LCPN	[7]
PS40	Tyrosine	LAT1	95 nm	TMZ; sorafenib	U87 MG	[27]
transformable block polymer	DHA	GLUT1	110 ± 1.54 nm	Ceramide; NFL-Pep	G422	[28]
bioengineered ferritin nanoparticles	ferritin; RGE peptide	TfR1; NRP-1	15.48 nm	STING agonists	GL261	[29]
disulfide–cross-linked polymeric	Angiopep-2 peptide	LRP-1	31 nm	Cas9/sgRNA	U87 MG	[31]
Ang-PEG-b-PGu; PEG-b-P(GuF)	Angiopep-2 peptide	LRP-1	110 nm	Cas9/sgRNA	U87 MG	[32]
MPC	choline analogue	nAChRs; ChTs	about 105 nm	antiPD-L1	LCPN	[33]
AuNPs	TPP peptide	Hsp70	about 45 nm	DOX	C6	[34]
PASP-g-PEI1800/dsb	2-amino-2-deoxy-D-glucose	GLUT1	92 nm	TMZ; siPD-L1	C6; GL261; U251	[36]
HSA	iRGD peptide	integrin αVβ3; neuropilin-1	about 100 nm	AMD3100	HF2303	[35]
fucoidan-based nanocarriers	fucoidan	P-selectin	80 ± 10 nm	vismodegib	SHH-MB	[37]
prodrug nanoparticles	Angiopep-2 peptide	LRP-1	55.27 ± 1.19 nm	Perifosine; TMZ; clodronate	U87 MG; GL261	[38]
liposome	D8 peptide; RI-VAP peptide	nAChRs; csGRP78	110–132 nm	Bortezomib	GL261	[39]
liposome	c(RGDyK) peptide	integrin αVβ3	about 110 nm	AL3810	U87 MG	[40]
liposome	quinolinium	–	54.3 ± 6.3 nm	photosensitizer	GL261	[41]
liposome	CDX peptide	nAChRs	122.5 nm	disulfiram/copper ion	U87 MG C6	[42]
liposome	Rg3	glucose transporter	66.12 ± 0.39 nm	PTX	C6	[43]
liposome	RGD peptide; lactoferrin	integrin αVβ3; LfR	135.8 ± 2.8 nm	Docetaxel	U87 MG	[44]
liposome	SS31 peptide	hyaluronic-acid−quercetin; mitochondria	162.2 ± 1.8 nm	DOX	U87 MG	[45]
liposome	glucose; TPPm	GLUT1; mitochondria	133.7 ± 4.29 nm	DOX; lonidamine	C6	[46]
liposome	Anti-EGFR	EGFR	71–80 nm	Cas9/sgRNA	005 GBM	[47]
liposome	Angiopep-2 peptide	LRP-1	100–200 nm	siRNA or Cas9/sgRNA	LN229	[48]
liposome	–	–	<200 nm	mRNA	dogs with spontaneous gliomas	[49]

Abbreviations: LDLR: low-density lipoprotein receptor-related protein; LRP: LDLR related protein; PS40: polyethylene glycol stearate; GrB: granzyme B; CpG: oligonucleotide; LAT1: L-type amino acid transporter 1; TfR1: transferrin receptor 1; DHA: Dehydroascorbic acid; GLUT1: glucose transporter 1; MPC: 2-methacryloyloxyethyl phosphorylcholine; nAChRs: nicotinic acetylcholine receptors; ChTs: choline transporters; TPP peptide: TKDNNLLGRFELSG; HSA: human serum albumin; Rg3: Ginsenoside Rg3; LfR: lactoferrin receptor; TPPm: triphenylphosphonium; EGFR: epidermal growth factor receptor.

### 3.2 Liposomes

Liposomes are spherical vesicles formed from phospholipid bilayers with physicochemical properties similar to those of cell membrane constituents. During synthesis, specific attributes such as lipid composition, charge, and size can be adjusted. Liposomes can transport both hydrophilic and hydrophobic drug molecules, shielding the drug from degradation and enhancing drug delivery to the tumor site [53,54]. Furthermore, liposomes exhibit stability in drug loading and pharmacokinetics, biocompatibility, low immunogenicity, and in vivo degradability [55]. Traditional liposomes face challenges in penetrating the BBB, functionalized and modified versions can serve as carriers for delivering drugs targeted at GBM.

Ligand-modified liposomes have the potential to penetrate the BBB. Researchers have developed an actively targeted liposomal nanoplatform using artesunate-phosphatidylcholine (ARTPC) liposomes incorporated with Apolipoprotein E (ApoE) peptides. This design enhances the nanoplatform's ability to penetrate the BBB and precisely target GBM (Fig. 2D) [50]. This approach effectively enhances treatment efficacy against GBM drug resistance following drug delivery. In addition, AL3810 is a reversible ATP-competitive inhibitor. When loaded into c(RGDyK)-modified liposomes, AL3810 could effectively across the BBB, inducing apoptosis in GBM cells and inhibiting tumor angiogenesis [40].

Liposomes have emerged as a promising tool for delivering siRNA or Cas9/sgRNA RNP across the BBB. Specifically, antibody-targeted liposomes, such as EGFR-targeted sgPLK1-cLNPs, have been developed to deliver Cas9/sgRNA RNP to GBM cells, enhancing targeted gene editing and potentially improving therapeutic outcomes for this aggressive brain tumor [47]. Recently, Zhao et al. reported a novel polymer-locked fusogenic liposome (Plofsome) capable of crossing the BBB to deliver siRNA and Cas9/sgRNA RNP to the cytoplasm of GBM cells (Fig. 2C). Plofsome is created by anchoring four-arm polyethylene glycol-catechol (4-arm PEG-oDPs) to the surface of Angiopep-2 modified liposomes. This polymer lock structure ensures that Plofsome fuses with tumor cells only after crossing the BBB and entering GBM tissue. Experimental results indicate that Plofsome effectively delivers siRNA or Cas9/sgRNA RNP to target tumor cells, demonstrating its potential as a therapeutic delivery system for GBM treatment [48].

Liposomal vaccines also have significant applications in the treatment of GBM. A study on the treatment of GBM with systemically administered mRNA aggregates (RNA-LPA) has achieved significant breakthroughs. Hector R et al. [49] developed an "onion-like" multilayer RNA-lipid particle aggregate (RNA-LPA) cancer vaccine, which has attracted significant attention. RNA-LPA significantly enhances the loading and immunogenicity of tumor mRNA antigens, demonstrating effective tumor suppression not only in mouse models but also in pet dogs with spontaneous late-stage GBM. In these dogs, RNA-LPA reprogrammed the TME and significantly extended their survival. A phase I clinical trial of RNA-LPA for GBM showed that after injection, the vaccine rapidly reprogrammed the patients' immune systems, triggering the release of cytokines/chemokines and immune activation/transport. This led to specific immune responses in GBM patients, indicating that RNA-LPA is a novel technology capable of reprogramming the TME while inducing a rapid and sustained cancer immunotherapy response.

Lipoproteins are natural liposomal nanoparticles found in the bloodstream, offering significant advantages for biomimetic drug delivery systems. By mimicking lipoprotein structures, these biomimetic liposomal delivery systems can evade monocyte phagocytosis, thereby extending their circulation time in the bloodstream. Compared to simple liposomes, these systems significantly enhance structural stability and biocompatibility [56]. Ras-activated GBM cells rely on macropinocytosis to deliver extracellular matrix proteins necessary for their growth and survival. To target these cells, researchers have developed lipoprotein biomimetic nanoparticles using recombinant high-density lipoprotein (ApoE-rHDL) to deliver siRNA precisely to GBM [57]. However, free HDL have limitations in tumor targeting and penetration, necessitating surface functional modifications. To address this, Wang et al. fused the tLyP-1 peptide onto the HDL surface, successfully enhancing BBB permeability and improving the targeting and accumulation of siRNA in GBM tissues [58]. Representative liposomes in GBM treatment are summarized in Table 1.

### 3.3 Cell membrane-camouflaged nanoparticles

**Fig. 3:**
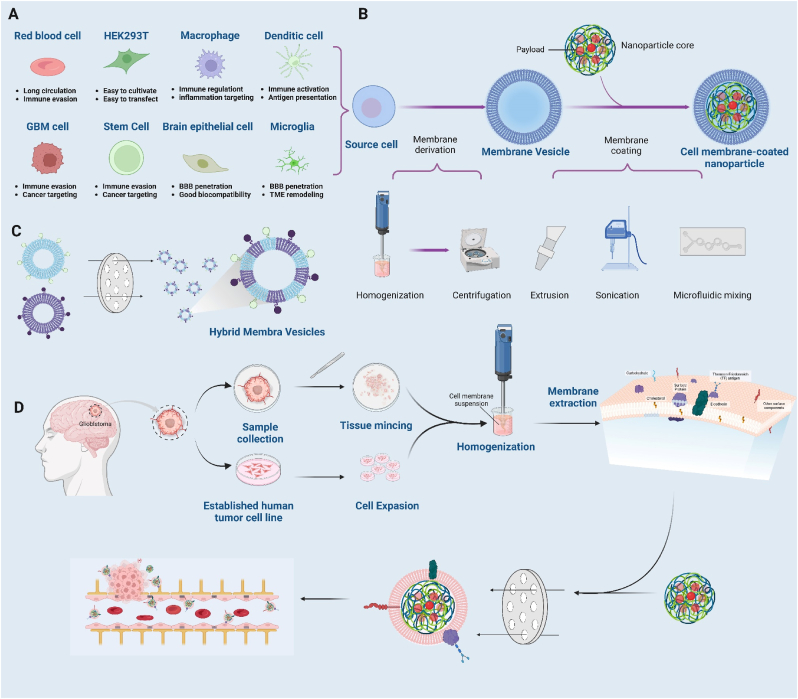
Fabrication of cell membrane-coated nanoparticles. (A) The selection of membrane sources for the fabrication of cell membrane-coated nanoparticles is guided by the unique properties of various cellular membranes; (B) These nanoparticles maintain the high drug loading capacities typical of traditional nanomaterials while enhancing biomimetic delivery through improved biocompatibility; (C) By integrating different cellular membranes, hybrid membranes are created, incorporating the advantages of each component; (D) Tumor heterogeneity significantly complicates drug delivery. However, leveraging homotypic recognition of tumor-specific membrane components could lead to innovative strategies. One promising approach might involve using patient-derived GBM cells to develop cell membrane-coated nanoparticle delivery systems tailored to individual patient profiles.

Cell membrane-Coated biomimetic nanoparticles (MNPs) are regarded natural multifunctional biomaterials. MNPs offer increased biocompatibility, hence substantially reducing the immunogenicity of nanoparticles to evade immune rejection. This property allows drugs to be gradually degraded or absorbed without excessive accumulation in the body, reducing the likelihood of excessive accumulation and decreasing the incidence of severe adverse reactions. Cell membranes sourced from erythrocytes, leukocytes, and cancer cells, among others, are referred to as "self-derived allies", significantly prolonging systemic circulation time to enhance tumor therapy (Fig. 3A) [59]. Highly biomimetic nanoparticles are developed by combining drugs for GBM treatment with nanocarriers and encapsulating them within cell membranes (Fig. 3B).

Red blood cell (RBC) membranes can evade clearance by the reticuloendothelial system. A multifunctional biomimetic nanoplatform was created through the surface functionalization of RBC membranes with Angiopep-2 [60]. This nanoplatform also contains pH-sensitive polymer loaded with DOX and Lexis. Lexis can transiently open the BBB, leading to improved BBB penetration and nanoparticle accumulation in the brain. Additionally, this group utilized RBC membranes decorated with ApoE peptide and pH-sensitive dextran nanoparticles to co-deliver ABT and A12, demonstrating significant suppression of GBM growth [61]. Drawing inspiration from the immune response activation in virus-infected cells, a nucleic acid nanogel coated with an RBC membrane and modified with M2pep and HA2 peptides, was designed to mimic a virus structure (Vir-Gel) (Fig. 2E). This delivery system effectively transports therapeutic miR155 to regulate the polarization of pro-tumor microglia and macrophages toward an anti-tumor phenotype. This process alleviates their immunosuppressive effects, enhancing the therapeutic impact on GBM [51].

Macrophage-camouflaged MNPs may enhance nanoparticle accumulation in chronic inflammatory tumor tissue. Building on this concept, Cao et al. engineered Angiopep-2-modified macrophage cell membranes to develop biomimetic nanoparticles that markedly inhibited GBM growth by inducing mitochondrial damage-induced iron death in tumor cells [62].

GBM cell membrane-camouflaged NPs can penetrate the BBB and selectively target homologous cancer cells, facilitated by the abundant presence of homotypic proteins on their surface (Fig. 3D). Researchers employed GL261 cell membranes to encapsulate small molecule Cu2-xSe loaded with indoximod (an inhibitor of indoleamine-2,3-dioxygenease in tumor) and JQ1(an inhibitor for reducing the expression of PD-L1), creating biomimetic nanoparticles capable of modulating the immunosuppressive microenvironment and inhibiting immune checkpoint functions effectively [79]. These NPs efficiently delivered drugs to the GBM site in the brain, altering the TIME and enhancing GBM immunotherapy outcomes. Another investigation also validated CD6 protein on the C6 cell membrane surface can bind to the leukocyte adhesion molecule (ALCAM) highly expressed on BBB endothelial cells, aiding in transporting nanoparticles across the BBB to the GBM site [77]. Nanosuspensions (NS) represent a new formulation for poorly soluble drugs, offering nearly 100 % high drug loading. However, they are readily identified and cleared by the reticuloendothelial system and mononuclear phagocytes upon entry into the bloodstream. To address this, researchers ingeniously constructed PTX nanosuspensions (NS) encased in C6 cell membranes and adorned them with D-type WSW peptide for precise drug delivery. PTX NS effectively facilitated penetration of BBB and targeted GBM, establishing a reliable platform for targeted drug delivery in GBM therapy [71].

Additionally, elevated lactate levels in GBM are reported, signifying lactate's crucial role in GBM progression. Building on this, researchers self-assembled hemoglobin (Hb), LOX, and CPPO-Ce6 into nanoparticles, which were further enveloped with U251 cell membranes to form a biomimetic cell system (M@HLPC). This system can easily penetrate the BBB, target GBM through homotypic recognition, and act as a co-therapeutic drug delivery agent based on lactate metabolism [80]. Zou et al. [83] developed an acid-sensitive biomimetic nanoplatform based on GBM cell membrane camouflage for co-delivery of TMZ and CDDP. Experimental findings showcased the efficiency of this biomimetic nanoplatform in drug loading, recognition, engulfment by homologous tumor cells, prolonged circulation in the body, effective BBB crossing, brain accumulation, and retention. Given the propensity of malignant tumors for brain metastasis, the brain metastatic tumor cell membrane-camouflaged nanocomposite can penetrate the BBB and transport therapeutics for brain tumor treatment [74].

Cell membrane hybridization has paved the way for developing various methodologies that combine the strengths of different cell membranes (Fig. 3C). The research leverages the unique properties of nanomaterials formed by combining cell membranes and mitochondrial membranes derived from GBM cells. This hybrid nanomaterial can specifically target GBM cells and their mitochondria [52]. This approach enhances the delivery of Gboxin, an inhibitor of oxidative phosphorylation, directly to the mitochondria within GBM cells. Furthermore, the encapsulation of Gboxin in this nanomaterial significantly improves its stability and pharmacokinetics in the bloodstream, leading to notable improvements in therapeutic effects against GBM (Fig. 2F).

Brain metastatic breast cancer cell membranes can breach the BBB, while GBM cell membranes possess a targeting affinity for GBM. Thus, nanocomposites coated with a hybrid fusion of these membranes inherit both the BBB permeability and the homologous targeting capabilities of GBM membranes from the parent cells [78]. By engineering an Angiopep-2 peptide-modified vesicle (ANG-TRP-PK1@EAVs) using a lipid extruder, researchers tackled challenges related to low natural exosome production, limited specific targeting capabilities, and exhibited non-cellular toxicity, stability, biocompatibility, and BBB penetration. The outcomes verified its efficacy as a potent drug carrier capable of transporting DOX across the BBB into GBM tissues. Representative cell membrane-camouflaged biomimetic nanoparticles in GBM treatment are summarized in Table 2.

**Table 2:** Summary of representative cell membrane-coated nanoparticles in GBM treatment.

Source of cell membrane	Ligand	Targeted site	Average Size	Therapeutic agents	Cell line	Ref.
RBC	M2pep and HA2 peptide	M2-microglial cells	about 131 nm	miR155	GL261	[51]
macrophage	Angiopep-2 peptide	LRP1	115.25 nm	saALOX15	LN229	[62]
macrophage	–	–	72.07 ± 2.62 nm	anti-NF-***κ***B peptides	GSCs	[63]
macrophage	ApoE	LRP-1; LRP-2; LDLR	about 140 nm	JQ1; Ce6	GL261	[64]
microglia	–	–	184.1 ± 1.01 nm	aPd-1; Mn-Porphyrin	GL261	[65]
bEnd.3	–	–	111.21 ± 4.76 nm	DOX	U87 MG	[66]
‵CAR T cell	4-1BB; CD3ζ intracellular	EGFR; CD133	about 107 nm	AIE-gens	U87 MG	[67]
U87 MG cell spheroids	cRGD	integrin αVβ4	149 ± 25 nm	Zn*x*Cd1−*x*S	U87 MG	[68]
U87 MG	–	–	about 250 nm	Gboxin; ZnGa_2_O_4_:Cr^3+^,Sn^4+^	U87 MG	[69]
U251	ApoE peptide	LDLR	about 188 nm	TMZ; lomeguatrib	U251	[70]
C6	DWSW peptide	–	about 169 nm	PTX	C6	[71]
C6	–	–	about 120 nm	pDNA	C6	[72]
GBM PDT cell	–	–	70–79 nm	AuNRs	GBM PDT cell	[73]
B16F10; 4T1	–	–	about 140 nm	indocyanine green	U87 MG	[74]
B16F10	–	–	about 106 nm	CS_2_; Bcl-2 siRNA	U87 MG	[75]
HEK293T	Angiopep-2 peptide	LRP1	about 220 nm	DOX	U87 MG	[76]
GL261	CD6	ALCAM	64 ± 3.5 nm	paroxetine; JQ1	GL261	[77]
MCF-7; U87 MG	–	–	88.89 nm	gambogic acid; indocyanine green	U87 MG	[78]
GL261	–	–	38.2 ± 6.8 nm	InDoximod; JQ-1	GL261	[79]
U251	–	–	118.7 nm	hemoglobin; lactate oxidase; CPPO-Ce6	U251; GL261	[80]
C6; DC	–	–	about 144 nm	docetaxel	C6	[81]
U87 MG; mitochondria	–	–	89.1 nm	Gboxin	U87 MG; X01	[52]
U251; RAW264.7; RBC	folic acid	FR	about 150 nm	lomitapide	U251	[82]

**Abbreviations:** GBM cell: U87MG, U251, C6, GL261; bEnd.3: brain microvascular endothelial cells; ALCAM: activated leukocyte cell adhesion molecule; B16F10: metastatic melanoma cell; GSCs: Glioma stem cells; DC: dendritic cells; AuNRs: cloak gold nanorods; FR: folate receptors; PDT: patient-derived tumor.

Polymeric nanoparticles offer numerous advantages, including a controlled-release profile, efficient drug-loading capabilities, and the ability to design their shape and size as needed. However, their limited biocompatibility presents a challenge. Liposomes, also known as artificial biomembranes, have good biocompatibility and promising applications, but they might encounter stability issues, and some liposomes might also provoke immune responses in the body. In contrast, cell membrane-coated nanoparticles, being natural constituents found in mammals, offer superior safety compared to artificial liposomes or polymeric nanoparticles, which may produce toxic compounds during their preparation and degradation processes. Given these considerations, developing biomimetic nanoparticles presents a compelling strategy for enhancing drug delivery to GBM (Fig. 3D).

## 4 Extracellular vesicles

**Fig. 4:**
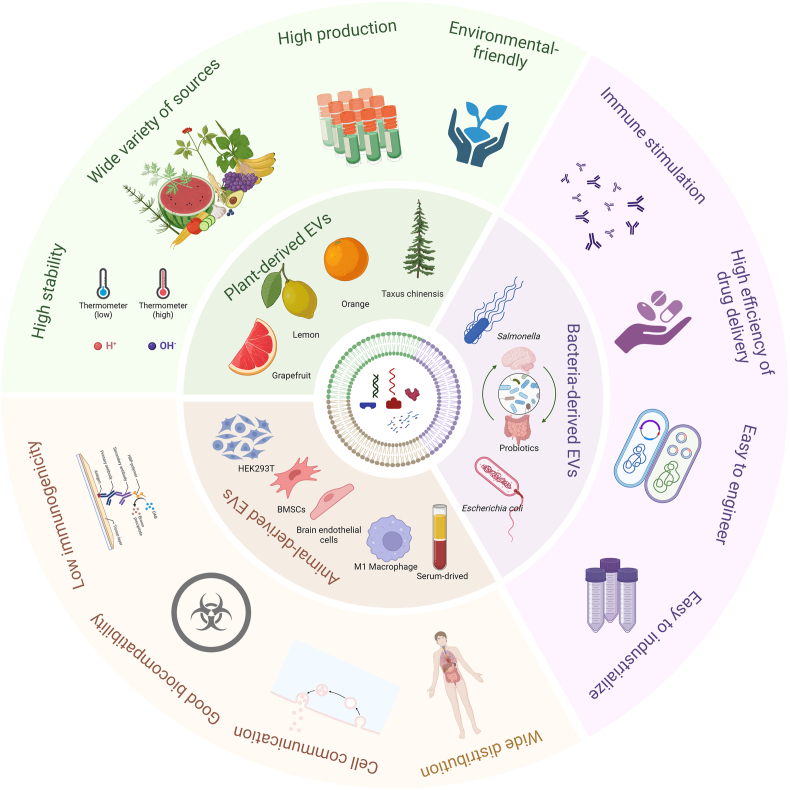
Overview of the specific properties of EVs derived from mammals, bacteria, and plants.

Almost all cells in nature including eukaryotes and prokaryotes release extracellular vesicles (EVs) as part of their normal physiological processes [84]. EVs function as essential intercellular communication carriers, facilitating the transportation of functional cargo between cells. They can be modified to effectively deliver various therapeutic payloads, such as siRNAs, antisense oligonucleotides, chemotherapeutic agents, and immune modulators, directing their delivery to specific targets. By utilizing the inherent properties of cell membranes, these biomimetic nanoparticles not only enhance biocompatibility and reduce immunogenicity but also offer improved targeting. However, natural EVs have limited capability to traverse the BBB, thereby diminishing their therapeutic efficacy. To overcome this limitation, engineering technology enables the integration of targeting units into the membrane surface of EVs, allowing therapeutics to across the BBB and accumulate in GBM tissues. This section provides a summary of the application of EVs in the treatment of GBM with a focus on the sources of EVs (Fig. 4).

### 4.1 Mammalian cell-derived EVs

EVs derived from mammalian cells (MEVs) are nanoscale membrane-bound vesicles crucial for intercellular communication and transport through paracrine, juxtracrine, and endocrine mechanisms. As intrinsic biological vesicles, MEVs have emerged as pivotal carriers in the brain.

To effectively traverse the BBB, EVs originating from brain cells are likely to exhibit brain-specific biomarkers. EVs derived from brain endothelial cells were identified as optimal candidates for drug delivery to GBM [85]. GBM-derived EVs possess a distinctive ability to communicate with their parent cancer cells [86]. Therefore, the utility of GBM-derived EVs loaded with selumetinib for GBM therapy, leveraging their inherent targeting capabilities [87]. GBM stem cell-derived EVs in cerebrospinal fluid can penetrate the BBB, facilitating GBM growth and migration. Thus, exogenous synthesis of EVs injected into the cerebrospinal fluid (CSF) may offer a potent strategy for GBM biotherapy [88]. Furthermore, the breast cancer-derived EVs can breach the intact BBB during brain metastasis, suggesting opportunities for designing drug delivery systems to transport therapeutics across the BBB [89]. However, caution is warranted when encountering tumor-associated macrophage or caner-derived EVs due to the potential involvement of their oncogenic cargo in advancing tumor progression [90,91]. For instance, the circNEIL3 could be encapsulated within EVs and transferred to infiltrating tumor-associated macrophages (TAMs), significantly influencing GBM progression and polarizing TAMs towards pro-tumor phenotypes [92].

**Fig. 5:**
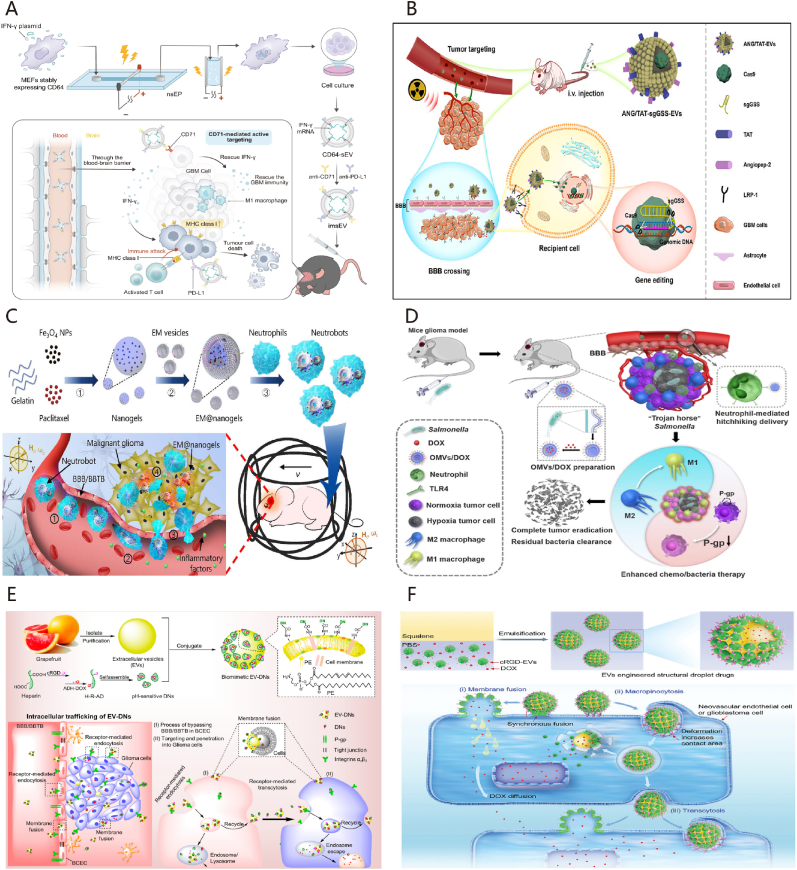
Strategies of EVs in the treatment of GBM. (A) The diagram illustrates the utilization of mRNA-loaded EVs to improve immunotherapy for GBM. Adapted with permission from Ref. [96]. Copyright 2023. Spring Nature. (B) The diagram depicts the use of engineered extracellular vesicles to deliver Cas9/sgRNA RNP complex for radiotherapy sensitization of GBM. Adapted with permission from Ref. [19]. Copyright 2023. American Chemical Society. (C) The diagram depicts the mechanism of magnetic field-controlled drug delivery targeting the GBM, achieved through neutrophil phagocytosis of magnetic nanogels enclosed within BEVs derived from *Escherichia coli*. Adapted with permission from Ref. [117]. Copyright 2021. American Association for the Advancement of Science. (D) The schematic illustration depicts a drug delivery system utilizing BEVs for the treatment of GBM. Adapted with permission from Ref. [119]. Copyright 2023 Elsevier. (E) The schematic illustrates a biomimetic delivery system based on PEVs, which bypass the BBB/BTB and penetrate GBM. Adapted with permission from Ref. [128]. Copyright 2021. American Chemical Society. (F) Schematic illustration of fruit-derived extracellular-vesicle-engineered structural droplet drugs for GBM chemotherapy. Adapted with permission from Ref. [20]. Copyright 2023. Wiley-VCH GmbH.

Mesenchymal stem cell (MSC)-derived EVs have demonstrate to inhibit GBM growth through the delivery of exosomal microRNAs (miRs) such as miR124 [93]. In addition, EVs-mediated miR124 delivery showed inhibiting M2 microglial polarization [94]; Verbascoside could enhance the expression of GBM-derived EVs containing miR-7-5p, thereby impeding GBM progression by blocking the EGFR/PI3K/Akt signaling pathway [95]. These results affirm that packaging miRNAs into EVs serves as a potent anti-GBM therapy deserving further clinical assessment [96] (Fig. 5A). Moreover, loading the Cas9/sgRNA RNP complex into EVs exhibited promising targeting of GBM tissue and yielded high efficiency in gene editing [19] (Fig. 5B).

MEVs possess robust cargo-loading and cargo-protective capabilities. The engineering of MEVs from different cell types, including embryonic stem cells (ESCs), brain endothelial cells, and GBM cells, among others, transforms them into targeted and versatile nanocarriers for drug delivery, encompassing drugs like TMZ, PTX, DOX, methotrexate (MTX), and other therapeutics [97]. Alvarez-Erviti et al. [98] firstly demonstrated the instance of receptor-mediated drug delivery to the CNS using RGV-modified EVs. Incorporation of target ligands can augment MEVs' capacity to traverse the BBB and confer them with GBM-targeting characteristics [99]. MEVs modified with tumor-targeting ligands, such as c(RGDyK) peptide and T7 peptide, exhibit enhanced abilities to navigate the BBB and target tumors [100,101]. Notably, heme oxygenase 1 (HMOX1) serves as a marker protein on the membrane surface of chemoresistant GBM cells, potentially serving as a recognition marker for GBM targeting [102]. MSC-EVs decorated with the HMOX1-specific peptide (HSSP) demonstrated enhanced targeting of TMZ-resistant GBM [103]. Furthermore, dual peptide-modified MEVs capable of multi-level targeting, with notable permeability to the BBB and precise targeting and tissue penetration of GBM [104]. Ye et al. [105] developed MTX-loaded MEVs modified with therapeutic and targeted peptides, marking a significant advancement in utilizing extracellular vesicles for selective target binding and therapeutic effects in brain tumor treatment.

MEVs play an important role in the immunotherapy of GBM. Exosome-mimetic vesicles created by extruding natural killer (NK) cells through filters exhibited enhanced cytotoxic effects on GBM cells compared to conventional NK cell-derived MEVs [106]. Furthermore, studies have indicated a direct correlation between GBM malignancy and the ratio of infiltrating M2/M1 macrophages in tumor tissues; a majority of these macrophages originate from the peripheral blood. Leveraging M1 macrophage-derived MEVs as carriers enables the accumulation of these vesicles at the GBM site due to the chemotactic properties of M1 macrophages [107]. This dual mechanism not only facilitates substantial macrophage presence at the tumor site but also modulates the immune environment of GBM by regulating macrophage phenotypes.

In terms of safety, MEVs represent a notable advancement in translational medicine, attributed to their low immunogenicity, biodegradability, and non-toxic nature [108]. Research has demonstrated that MEVs can transmit a "don't eat me" signal through CD47, CD55, and CD200; shielding them from phagocytosis or elimination [109]. While MEVs exhibit the capacity to evade immune surveillance and cross the BBB, several studies have indicated that when administered intravenously, MEVs predominantly accumulate in the liver or spleen, with only a minority of MEVs reach the tumor site [110]. Numerous uncertainties persist in the exploration of MEVs, emphasizing the critical need for a thorough and precise characterization of MEVs, understanding the heterogeneity of MEVs, and the evolution of their cargo and function [111]. The outcomes of these preclinical studies suggest a significant potential for MEVs as therapeutic agents.

### 4.2 Bacterial extracellular vesicles

Bacterial extracellular vesicles (BEVs) are lipid nanovesicles secreted by bacteria, serving as a significant tool for bacterial communication with host cells and modulation of the host immune response to evade immune surveillance [112]. BEVs loaded with therapeutic molecules are becoming a promising option due to their distinctive nanoscale structures, stable loading capacity, good biocompatibility, and ease of modification. The rapid proliferation and robust adaptability of bacteria allow for industrial production to generate abundant sources of BEVs for clinical use. Similar to mammalian EVs, BEVs can be engineered to target GBM across the BBB. Furthermore, BEVs harbor numerous virulence factors and pathogen-associated molecular patterns, which possess intrinsic anti-tumor properties and can stimulate the body's immune response to combat tumors [113].

Although BEVs have been widely utilized in tumor research, the existing literature regarding GBM treatment is scarce. Chen et al. [114] conducted a study where they effectively synthesized a complex of two specific nanoparticles, BEVs from *E. coli* and gold nanoparticles (AuNPs). This novel combination, along with radiotherapy, exhibited radiosensitizing and immunomodulatory effects that significantly enhanced the treatment of GBM. Another research team developed a brain-targeted drug delivery system, known as dBEVs@NPs, based on the removal of lipopolysaccharide (LPS) from the biocompatible *EC-K1* bacterial outer membrane. This system leverages the interaction between the outer membrane functional protein (OmpA) and the BBB endothelial cell receptor gP96 to efficiently across the BBB and deliver therapeutics into the brain, demonstrating promising targeting abilities for efficient drug delivery [115]. Based on this study, He et al. [116] designed an LPS-free BEVs camouflaged biomimetic nanoreactor tailored to target tumor cells, achieving quadruple synergistic therapy for orthotopic GBM.

The integration of BEVs with nanomaterials can confer a "camouflage" effect, enhancing the phagocytosis of drug-carrying particles by neutrophils and improving the biocompatibility of the drug delivery system to prevent drug leakage (Fig. 5C) [117]. Studies have indicated that certain anaerobic bacteria, like *Salmonella*, can selectively colonize within hypoxic regions of tumors [118]. Mi et al. [119] illustrated that a particular strain of *Salmonella (S.t-ΔpGFLaB)* exhibited specific targeting towards GBM sites and facilitated the recruitment of neutrophils through the BBB, enabling the delivery of chemotherapeutic drugs encapsulated within BEVs into the tumor site (Fig. 5D). Moreover, *Salmonella* induced the transformation of macrophages from M2 to M1 phenotype and down-regulated the expression of P-glycoprotein, thereby achieving targeted therapy for GBM.

Evidence suggests that BEVs released by gut microbiota play a crucial role in the brain-gut axis. These BEVs can traverse the BBB and relay diverse signals to the brain via their rich array of proteins and nucleic acids, exerting regulatory functions on brain cells such as neurons, astrocytes and microglia [120]. Studies have demonstrated that metabolites from intestinal commensals can activate the aromatic hydrocarbon receptor (ARH) on astrocytes and tumor-associated macrophages (TAMs), thereby limiting CNS inflammation and influencing immune evasion mechanisms in GBM [121,122]. Therefore, BEVs derived from specific probiotics exhibit varying therapeutic effects in a range of neurological disorders.

### 4.3 Plant extracellular vesicles

Plant extracellular vesicles (PEVs) are membranous structures originating from plant cells, playing pivotal roles in diverse physiological and pathological processes. Referred to as exosome-like nanoparticles (ELNs) due to unclear biogenesis and secretion pathways [123]. PEVs are known to harbor various components, such as proteins, enzymes, and siRNAs, crucial for maintaining homeostasis, facilitating intercellular communication, and bolstering immune defense responses [123,124]. Their therapeutic potential has garnered significant interest, especially considering that PEVs are abundant, cost-effective, and easily accessible compared to their mammalian counterparts, offering a promising alternative to address technical challenges associated with mammalian vesicles [125].

Research on utilizing PEVs for treating GBM remains limited, although some studies date back a decade. Nonetheless, earliest attention has been directed towards the therapeutic potential of PEVs. Noteworthy studies by Wang et al. have showcased the ability of grapefruit-derived EVs (GEVs) to transport an anti-Stat3 inhibitor (JSI-124) to the brain intranasally, effectively inhibiting GL26 tumor growth [126]. Further investigations by the same group revealed that GEVs, especially when coated with folic acid, exhibit enhanced targeting efficiency towards folate receptor-positive GL-26 brain tumors, making them highly suitable vectors for delivering siRNA [127]. Niu et al. [128] developed a biomimetic delivery system by attaching pH-sensitive nanoparticles (NPs) onto GEVs, leveraging cRGD peptide modification to facilitate bypassing of the BBB/BTB and infiltration into GBM tissues (Fig. 5E). This innovative strategy significantly increases payload capacity and targeting specificity compared to conventional EVs encapsulation methods, as evidenced by the heightened accumulation of EV-DNs within GBM tissues, bolstering the efficacy of EVs-based drug delivery systems. Departing from conventional approaches employing PEVs solely as drug carriers, this research group further introduced a versatile drug delivery system, termed PEV-engineered structural droplet drugs (ESDDs). By utilizing fruit-derived EVs as particle surfactants and engineering the self-assembly of cRGD-modified EVs at the squalene–PBS interface, the ESDDs demonstrate remarkable efficiency in traversing the BBB/BTB and deeply penetrating GBM tissues through a flexible delivery mechanism involving deformation-amplified macropinocytosis and membrane fusion. This innovative approach surpasses the capabilities of cRGD-modified PEVs, significantly enhancing antitumor efficacy against GBM (Fig. 5F) [20].

PEVs serve as natural carriers for biological drugs, efficiently protecting and transporting hydrophilic or hydrophobic substances to particular locations [129]. By modifying their surface membranes with suitable ligands, the accuracy of their targeting can be improved, a fact supported by various studies demonstrating the harmless properties of PEVs obtained from edible plants. Moreover, plants have the potential to act as "nanofactories," sustainably producing medical PEVs on a large scale. Despite the current scarcity of brain drug delivery studies utilizing PEVs, the advantages of PEVs as natural nanocarriers make them indispensable in GBM therapy.

Historically, research on EVs as drug delivery vehicles has predominantly centered on vesicles of mammalian origin. However, mammalian-derived EVs are hindered by the drawbacks of high culture conditions, elevated cost, and limited extraction yield [54]. In recent years, there has been a growing body of research on EVs derived from bacteria and plants as drug delivery vehicles. Both BEVs and PEVs offer the advantage of being capable of large-scale production and easily engineered. Nevertheless, the emphasis on GBM treatment should prioritize treatment effectiveness. Additionally, irrespective of the EVs' origin (mammalian, bacterial, or plant), safety considerations must be upheld. Representative EVs derived from mammalian, bacterial and plant in GBM treatment are summarized in Table 3.

**Table 3:** Summary of representative extracellular vesicles in GBM treatment.

	Source of EVs	Ligand	Target	Average size	Therapeutic agents	Cell Line	Ref.
MEVs	M1 macrophage	–	–	about 100 nm	AQ4N; CPPO; Ce6	U87 MG; G422	[107]
	HEK293T	Angiopep-2 peptide and TAT peptide	LRP1	125 ± 40 nm	Cas9/sgRNA	LN229	[19]
	ReN	c(RGDyK))	integrin αVβ3	100–250 nm	siPDL1	GL261	[130]
	ESCs	c(RGDyK))	integrin αVβ4	about 75 nm	PTX	U87 MG	[100]
	HEK293T	Angiopep-2 peptide and TAT peptide	LRP1	120 ± 20 nm	Dox	U87 MG	[104]
	BMSCs	HSSP peptide	HMOX1	70 nm	TMZ; siRNA	U251	[103]
	HEK293T	anti-CD71; anti-PD-L1	CD71; PD-L1	about 120 nm	IFN-γ mRNA and anti-PDL1	GL261	[96]
	BEnd.3	transferrin	TfR1	122.6 ± 7.2 nm	TPP-Ce6	U87 MG	[85]
	BV2	redox-response oligopeptide	–	about 100 nm	DOX	U87 MG	[131]
	chimeric exosomes	–	–	40–160 nm	cGAMP	GL261	[132]
	BMSCs	GRE peptide	NRP-1	about 80 nm	nanocatalysts	C6	[133]
	serum	TfR (Bind with free Tf in the blood)	TfR	111.6 ± 12.1 nm	transcription 3; CpG peptide	GL261	[134]
	HEK293T	Angipep-2 peptide	LRP1	about 200 nm	DOX	U87 MG	[76]
BEVs	*Escherichia coli*	neutrophils-mediated	inflammatory	about 105 nm	PTX and Fe_3_O_4_	G422	[117]
	*Escherichia coli*	–	–	42 nm	AuNPs	G261	[114]
	*Salmonella*	neutrophils-mediated	TIM	94 nm	DOX	C6	[119]
	*Escherichia coli K1*	OmpA	gp-96	119.3 nm	DOX	231Br	[115]
	*Escherichia coli K1*	OmpA	gp-97	188.6 ± 12.5 nm	Glucose oxidase; Cu9S8; AQ4N	GL261	[116]
PEVs	grapefruit	folic acid	FRs	186.8 nm	JSI-124; PTX	GL26	[126]
	grapefruit	folic acid	FRs	87.2 nm	miR17	GL27	[127]
	grapefruit	cRGD	integrin αVβ3	166 ± 4 nm	DOX	LN229	[128]
	lemon	cRGD	integrin αVβ4	0.5 μm or 2.5 um	DOX	GL261	[20]
	citrus fruit	Angiopep-2 peptide	LRP1	195.03 ± 6.77 nm	DOX; Vitamin C; Vitamin E; hesperidin;	GL261	[135]

**Abbreviations:** CPPO: hydrophobicbis (2,4,5-trichloro-6-carbopentoxyphenyl) oxalate; Ce6: chlorin e6; siPDL1: siRNA against PD-L1; ESCs: embryonic stem cells; MSC: mesenchymal stem cells; BMSC: bone marrow MSC; PTX: paclitaxel; HMOX1: heme oxygenase-1; HSSP: HMOX1 specific short peptide; TPP: triphenylphosphonium; BV2: mouse microglial cell; cGAMP: 2′3′-cyclic guanosine monophosphate–adenosine monophosphate; TIM: tumor immune microenvironment; OmpA: Outer membrane protein A; FRs: folate receptors.

## 5 Neurotropic viruses

The advancement of gene editing therapies has led to the widespread application of gene editing agents in treating GBM [136]. Neurotropic viruses, displaying an affinity for nerve tissue, have naturally evolved mechanisms to breach the BBB and convey nucleic acid cargos directly to tumor cells [11]. Consequently, these viruses can be harnessed as carriers to transport gene editing agents and other therapeutic substances across the BBB, specifically targeting GBM. Various neurotropic viruses have been engineered to achieve efficient BBB penetration, making them promising tools in the fight against this aggressive brain tumor.

### 5.1 Adeno-associated virus delivery

The adeno-associated virus (AAV) is non-enveloped approximately 25 nm in size, which can be engineered for efficient payload delivery to the brain [137]. Renowned for its distinctive biological and biophysical properties, AAV has garnered widespread interest in gene therapy, demonstrating safety and efficacy in both preclinical and clinical realms [138]. The AAV's protective protein shell, along with its genetic material inside, can be adjusted to improve targeting of specific tissues and control the expression of genes, as mentioned in Ref. [139]. Currently, AAV is the most frequently used viral vector in gene therapies, effective in delivering macromolecular therapeutics in the form of DNA [140].

**Fig. 6:**
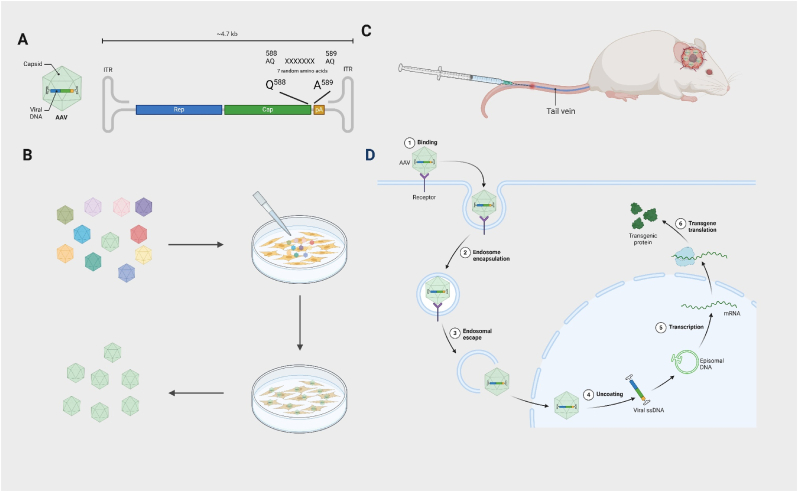
Engineering AAV variants for systemic delivery to the GBM. (A) Building an AAV capsid DNA library by inserting seven random amino acids between VP1 residues 588 and 589; (B) Screening for AAV variants with affinity for GBM at the cellular level; (C) Administer the AAV virus library via intravenous injection in animal models, selecting for variants capable of penetrating the BBB and target GBM tissue; (D) Upon penetrating the BBB, the AAV binds to specific receptors on the surface of GBM cells and enters them, becoming enclosed within endocytic vesicles. Subsequently, the AAV escapes into the cell nucleus where its single-stranded DNA unpacks and converts into double-stranded DNA. This DNA undergoes transcription and translation processes, ultimately leading to the production of protein molecules capable of treating GBM.

Natural AAV capsid serotypes, such as AAV9, AAVrh.10, and AAVHSC, exhibit a degree of BBB permeability, with augmented penetration achieved through customized coat protein modifications in AAV mutants like AAV-AS, Anc80L65, AAV.PHP.B, AAV.CAP-B10, and AAV-F, among others [[141], [142], [143], [144], [145], [146], [147]]. In particular, AAV capsid protein modification combined with directed evolution in vitro and in vivo holds promise in optimizing tissue tropism [148]. Examining modifications in the AAV9 capsid, researchers have successfully inserted a random 7-mer peptide between amino acids 588 and 589 in the hyper-variable region VIII—a deliberate approach that enables the variable peptide sequence to be visible on the surface of the capsid (Fig. 6) [141,[149], [150], [151]]. The evolving landscape suggests the imminent creation of more AAV variants tailored to breach the BBB.

The intravenous delivery of AAV9 encoding therapeutic genes presents a compelling strategy for GBM treatment [152,153]. Investigations indicate that systemic administration of AAV9-IFN-β proves more efficacious in combating multifocal GBM than localized injections [152]. Recognizing the oncogenic role of HUWE1 in GBM, researchers have developed an AAV9 dual-vector system for administering dCas9-VP64 to induce endogenous HUWE1 expression in GBM cells, demonstrating promising anti-GBM efficacy [154]. Noteworthy advancements include the creation of AAV.CPP.16 by Bei et al., achieved through a rational design strategy for the AAV9 viral capsid [141]. This novel construct exhibited enhanced efficiency in systemic gene delivery for BBB traversal in mice and non-human primates, with a focus on delivering anti-cancer drugs such as PD-L1 or Herpes simplex virus 1 thymidine kinase (HSV-TK1) to GBM cells. Consequently, AAV.CPP.16 showcased targeted tumor cell eradication, significantly prolonging the survival of mice with GBM. By leveraging a specialized AAV vector targeting brain endothelial cells, LIGHT expression within the tumor blood vessels of GBM-bearing mice was facilitated, leading to the development of tumor-associated High Endothelial Venules (HEVs) and Tertiary Lymphoid Structures (TLS). This orchestrated cascade enhanced T cell activation in the tumor, effectively prolonging the lifespan of GBM mice resistant to αPD-1 therapy [155]. Treating GBM not by crossing the BBB but by repairing it. Martin et al. employed a single “hit-and-run” intravenous AAV-PHP.eB gene delivery approach, focusing on reinforcing BBB integrity rather than breaching it. Through genetic engineering of developmental BBB regulators into BBB-protective Wnt activators, GBM expansion was successfully mitigated [156].

Additionally, researchers have pioneered a targeted hybrid delivery system merging AAV and phage vectors, incorporating genetic components from AAV and single-stranded M13-derived bacteriophages decorated with tumor-targeting ligand peptide sequences, effectively pinpointing GBM cells [157]. Specifically, aPD-1 encoding HER2-AAV underwent genetic engineering incorporating a HER2-specific protein onto the viral surface, imparting specificity towards HER2+ GBM cells. Regrettably, no intracranial anti-PD-1 effects were observed upon systemic HER2-AAV administration, suggesting a limitation in crossing the BBB solely by HER2-AAV [158]. This emphasizes the necessity for alignment between receptor and ligand expressions on BBB cells for successful transmission.

AAV has advanced to clinical trials for various CNS diseases, including spinal muscular atrophy (SMA) and mucopolysaccharidoses (MPS), demonstrating effective therapeutic outcomes [159]. However, significant challenges remain in using AAV to treat GBM. Effective gene transduction and symptom relief in the brain often require large quantities of viral vectors. Repeated intravenous injections, however, can lower the efficacy of these vectors due to the presence of anti-AAV antibodies. Furthermore, the natural liver tropism of AAV leads to preferential accumulation in the liver, increasing the risk of hepatotoxicity. The ectopic expression of target genes in peripheral tissues also poses additional safety concerns. Nevertheless, the clinical success of AAV gene therapy in CNS diseases has stimulated efforts to address these challenges. As a result, the future of AAV gene therapy for GBM holds significant promise.

### 5.2 Other viruses and virus-like particles

Lentiviruses have a larger packaging capability than AAV and originate from the Human Immunodeficiency Virus (HIV), with the distinct capacity to infect both dividing and non-dividing cells and merge into the genome [160]. In a bid to circumvent impacts on normal cells, lentivirus is predominantly used for intratumoral injections for GBM or by ex vivo modification of patients’ cells [161]. Adenoviruses, characterized by their non-enveloped, double-stranded DNA structure, have been widely examined in preclinical and clinical trials for GBM therapies. A phase II clinical trial demonstrated the antitumor effectiveness of intra-arterial replication-deficient adenovirus mutant HSV-TK1 combined with systemic ganciclovir in recurrent GBM patients [[162], [163], [164]]. Although viral vectors display limited ability to penetrate the BBB and infiltrate tumor parenchyma, neural stem cells have been identified as capable of BBB penetration, infiltration into tumor zones, growth around tumor margins, and migration within brain tissue to target GBM cells. Employing neural stem cells for AV delivery is a novel brain-targeted strategy with lower immunogenicity [163]. Additionally, viruses like Newcastle disease virus, parvovirus, and rhinovirus possess the potential to cross the BBB, enabling intravenous delivery to reach intracranial tumor sites [[165], [166], [167]].

Virus-like particles (VLPs) are assembled from one or multiple structural proteins of different viruses, mimicking virus particles in overall structure but devoid of viral nucleic acids, thus circumventing replication and genomic integration risks [168]. Due to their exceptional safety profile and resemblance to viral entities, VLPs are used for nanovesicles for delivering diverse therapeutics such as nucleic acids, small molecules, and proteins [169]. Notably, genome-free VLPs have been crafted for HSVI-TK delivery, inducing efficient eradication of human GBM cells in conjunction with ganciclovir [170]. Furthermore, VLP surfaces can be tailored for site-specific targeting and evasion of phagocytic clearance [171]. In innovative approaches, researchers have engineered bacteriophage Qβ particles into RNA interference (RNAi) therapeutics to enhance radiotherapy efficacy against GBM by impeding DNA repair. Subsequent TAT peptide conjugation enhances RNAi penetration, culminating in robust gene silencing [172].

## 6 Challenges and future perspectives

### 6.1 Challenges

#### 6.1.1 Heterogeneity and drug resistance in GBM

GBM exhibits extensive cellular and genetic heterogeneity both among patients and within individual tumors over time and space. This heterogeneity may result from genetic mutations and chromosomal structural variations [173]. Even tumors of the same type can have vastly different genetic mutations and pathological features, posing significant challenges to the development of precise drug delivery systems. Within a single patient, GBM can display diverse tumor cell populations, complicating efforts to eradicate all cancer cells with a single treatment approach. This is particularly challenging for drug delivery systems, as a single receptor-mediated method may not target all tumor cell populations.

During the growth of GBM, this heterogeneity can contribute to treatment resistance [174]. GBM cells often upregulate drug efflux pumps, such as P-glycoprotein, to reduce the intracellular accumulation of chemotherapeutic agents, thereby diminishing their efficacy. Additionally, different tumor cell populations exhibit varying resistance mechanisms, resulting in different sensitivities to the same therapeutic agent. Moreover, a small population of cancer stem cells within GBM often shows inherent resistance to chemotherapy and radiotherapy, potentially leading to tumor recurrence post-treatment. Therefore, drug resistance presents another significant challenge to the effectively delivery of therapeutics for GBM.

#### 6.1.2 Limitations of preclinical studies

Although substantial evidence supports the efficacy of drug delivery systems in precisely targeting GBM, the majority of studies rely on human-derived GBM cell lines (e.g., U251, U87, T98) rather than patient-derived tumor cells to establish orthotopic xenograft models (Table 1, Table 2, Table 3). Additionally, the frequent use of immunodeficient Balb/c nude mice does not accurately replicate the performance of drug delivery systems within a normal immune environment. This discrepancy may result in rapid drug clearance by the immune system, impeding the evaluation of glioblastoma's immune evasion mechanisms and their influence on therapeutic efficacy. Furthermore, many studies utilize fewer than ten mice, limiting the statistical power and reliability of their findings.

To overcome these limitations, we recommend increasing the number of animals in preclinical studies to improve statistical robustness. Moreover, collaboration between basic researchers and clinicians should be actively encouraged to facilitate the analysis of patient-derived tumor specimens and the establishment of xenograft models using patient-derived GBM cells. Shifting away from dependence on a few representative cell lines will better capture the complexity of GBM and yield more compelling and clinically translatable results.

### 6.2 Future prospectives

To overcome the challenges of GBM tumor heterogeneity and drug resistance while ensuring that drug delivery systems persist in terms of delivery efficiency, precision targeting, and safety, regulating drug release dynamics at tumor sites is essential for improving therapeutic outcomes. The GBM microenvironment is characterized by weak acidity, hypoxia, elevated protease levels, and heightened cytoplasmic glutathione (GSH), all of which serve as endogenous stimuli for localized drug release. Moreover, these systems can be designed to respond to external triggers such as light, heat, ultrasound, and magnetic fields. Each drug delivery approach offers unique advantages and limitations. Therefore, integrating diverse systems holds significant potential for the development of an efficient brain drug delivery platform.

#### 6.2.1 Double ligand targeting

Dual ligand-modified nanocarriers represent an innovative drug delivery system for GBM. GBM cells exhibit significant heterogeneity, making the binding of a single ligand to a receptor not universally suitable for all tumor cells in patients. The application of chimeric antigen receptor (CAR) -T immunotherapy in tumors such as GBM often involves a dual-targeted treatment approach to address antigen immunity [175]. For example, utilizing CAR-T cells targeting both EGFR and IL13Rα2 demonstrated effective inhibition of tumor growth in a phase I clinical trial, showing superior outcomes compared to single-target therapies [176]. Moreover, the effectiveness of a dual-targeted delivery strategy in the GBM-specific drug delivery system has been confirmed in preclinical studies.

To establish a dual-targeted drug delivery system for GBM, the following strategies are employed. Nanomaterials are functionalized through chemical modification by attaching two or more targeting ligands (such as peptides, proteins, and drug molecules) to the material surface. Additionally, tumor cell membranes are isolated to perform multi-targeting experiments utilizing the mutual recognition properties of membrane proteins. For instance, researches has explored the fusion of GBM cell membranes with mitochondrial membranes to achieve dual-targeting of tumor cells and their mitochondria [52]. In terms of EVs and neurotropic viruses, gene engineering techniques can be utilized to express multiple targeting peptides or proteins on the cell membrane or virus surface, enabling dual-targeting capabilities. Furthermore, enhancing the precision of drug delivery can be achieved by modifying the surface of nanocarriers with specific ligands and cell-penetrating peptides (CPPs) as a key strategy [177]. For example, functional EVs modified with Angiopep-2 peptide and TAT peptide exhibit efficient BBB crossing and GBM targeting [19,104]. TAT easily penetrates the cell membrane, overcoming Angiopep-2 receptor saturation, which enhances BBB permeability and promotes tumor penetration. CPPs have been successfully combined with targeting peptides, such as vascular endothelial-cadherin-derived peptide (pVEC), pentapeptide QL [178], pentapeptide QL [179] and RVG peptide [180], for brain-targeted drug delivery.

#### 6.2.2 Cell mediated delivery

The cell-mediated drug delivery system utilizes the unique physiological functions of cells to transverse the BBB and deliver therapeutics targeted to GBM. Cells can also be engineered to spontaneously produce therapeutics or act as Trojan horses carrying nanoparticles encapsulating drugs [181]. Cell-based drug delivery systems exhibit advantages such as prolonged circulation in the body, targeting specificity, and biocompatibility.

Immune cells have a strong tendency to migrate towards inflammation and can actively deliver drugs to inflammatory sites. Neutrophils can traverse the BBB and transport drugs into inflamed sites such as GBM. Consequently, they can be preloaded with nanomaterials before injection in vitro, or the nanomaterials can be designed to be specifically taken up by neutrophils in the bloodstream before accumulating in GBM [182]. Neutrophils have been developed as drug carriers, loaded with PTX nanoparticles for targeted delivery to GBM lesions, hence elevating drug concentration in the tumor area [183,184]. CAR-neutrophils, engineered to provide the optimal anti-tumor performance, are also designed to deliver therapeutics specifically and noninvasively to target GBM, without inducing additional inflammation at the tumor sites [185].

The application of CAR-T cells in the treatment of GBM has been limited by the BBB and BTB. Recently, a programmable navigation-guided cytotoxic T cell has significantly expanded the possibility of T cells entering the brain to eliminate GBM [186]. These engineered T cells initially seek out the highly specific protein brevican (BCAN) within the brain's extracellular matrix. Subsequently, they identify GBM-specific proteins, such as the GBM-associated antigen EphA2. It is only after accurately targeting the tumor that they activate the expression of CAR genes or other genetically encoded payloads to destroy the proliferating tumor. Studies show that this sophisticated design enables CAR-T cells to efficiently penetrate the mouse brain and eliminate primary GBM while avoiding peripheral or systemic toxicity.

Various other cells, including macrophages, mesenchymal stem cells, olfactory ensheathing cells, and dendritic cells, have also been confirmed to mediate drug delivery for GBM [[187], [188], [189], [190], [191], [192]]. Leveraging these cell-based delivery systems holds significant potential to enhance the precision and efficacy of GBM treatment.

#### 6.2.3 Stimuli-responsive drug release

Drug delivery systems responsive to internal or external stimuli offer a viable approach for achieving targeted accumulation and controlled release of anticancer agents at GBM lesions, ultimately enhancing drug safety and efficacy.

Endogenous regulation of drug release hinges on disparities between the tumor microenvironment and normal tissue. The Warburg effect observed in GBM cells leads to substantial acidification of extracellular fluid in GBM cells (pH 6.5–6.8) in contrast to normal cells (pH 7.3–7.4) [193]. To maintain redox balance, tumor cells typically exhibit levels of reactive oxygen species (ROS) that are 100 times higher and concentrations of glutathione (GSH) that are 7–10 times greater than those in normal cells [194]. As a result, many studies have developed drug release strategies that respond to factors such as pH, ROS, and GSH within the tumor microenvironment, ensuring precise drug release in GBM cells while minimizing toxic effects on normal cells due to the unique microenvironmental conditions [31,195]. For instance, ROS-responsive nanocarriers swiftly respond to elevated ROS levels in GBM cell mitochondria, liberating Gboxin to selectively accumulate in GBM mitochondria, and impeding ATP synthase activation to trigger apoptosis pathways, thereby effectively inhibiting tumor growth [52].

External stimuli-responsive drug release can be achieved through the use of light, ultrasound, or magnetic manipulation. Application of appropriately wavelength-specific light sources, like ultraviolet (UV) and Near-Infrared (NIR), to photosensitizer-containing polymers can induce conformational changes, leading to drug release [196]. Additionally, cellular uptake of photosensitizers under specific light sources prompts photochemical reactions generating reactive oxygen species, singlet oxygen, or thermal energy, selectively damaging cells and serving therapeutic objectives, known as photodynamic therapy (PDT) and photothermal therapy (PTT). The effectiveness of natural killer cell membrane-coated aggregation-induced emission (AIE) nanorobots with Near-Infrared II fluorescence properties in targeting GBM cells has been demonstrated [197]. These nanorobots significantly inhibit tumor growth upon external Near-Infrared light irradiation. Ultrasound serves as a mechanical pressure wave that enhances BBB permeability, facilitating drug release and precise sonodynamic therapy (SDT) for GBM [195]. Magnetic nanoparticles, under magnetic field guidance, can be directed to specific sites, exemplified by researchers employing a magnetic helmet to concentrate magnetic nanoparticles in GBM tissue for ferroptosis treatment [117,198,199].

#### 6.2.4 Co-delivery strategy

To enhance BBB penetration, researchers have undertaken strategic approaches by integrating extracellular vesicles, nanomaterials, and viruses to capitalize on their individual strengths. Nanomaterials not only serve as the core of the delivery system but can also form conjugate systems with extracellular vesicles. Li et al. [199] devised a novel composite therapeutic platform by amalgamating Angiopep-2 peptide-modified engineered EVs with magnetic nanoparticles using antigen-antibody interactions. This combined approach leverages the magnetic targeting capabilities of magnetic nanoparticles, as well as the BBB-penetrating potential and siRNA encapsulation properties of EVs.

One of the primary challenges encountered with intravenous AAV injection is the presence of preexisting neutralizing antibodies (NAbs) that bind to free AAV, hindering efficient gene transduction and diminishing therapeutic efficacy. Extracellular vesicle-encapsulated AAVs (EV-AAVs) have emerged as a superior gene delivery vector for CNS targeting, offering heightened NAb resistance compared to free AAVs [200]. Moreover, engineered extracellular vesicles can facilitate AAV BBB penetration. Through optimization of a size exclusion chromatography (SEC) procedure for generating and isolating brain-targeting EV-AAVs, researchers demonstrated their ability to traverse the BBB following intravenous administration [201].

The heterogeneity in GBM presents a significant challenge to effective tumor therapy. It is imperative to explore synergistic approaches targeting not only GBM but also tumor-associated macrophages (TAMs) and glioma stem-like cells (GSCs), employing various anti-tumor mechanisms to enhance treatment outcomes [38]. The outlined delivery systems enable the simultaneous delivery of different theraputics to attain synergistic effects for the treatment of GBM. Zhang et al. [38] harnessed drug properties to devise prodrug nanoparticles containing perifosine, TMZ, and clodronate, aiming to address GBM heterogeneity and elicit potent anti-GBM effects. Granzyme B (GrB), a serine protease acting as a crucial mediator in natural killer cells and cytotoxic T lymphocytes, exerts tumor cell destruction by targeting mitochondria. Co-administration of the immunoadjuvant CpG oligonucleotide (CpG) boosts GrB immunotherapy. Therefore, Wei et al. developed ApoE peptide-functionalized polymersomes for the co-delivery of GrB and CpG, facilitating BBB penetration and prompting robust ICD [7].

As diverse drug co-delivery strategies evolve, further investigations are warranted to determine optimal ratios, synergistic effects, and potential side effects accompanying simultaneous delivery of multiple drugs.

## 7 Status of clinical trials

Preclinical research is a vital step in the drug development process, involving a comprehensive evaluation of a drug's safety, efficacy, and pharmacokinetic properties in animal models and in vitro systems to ensure the safety and effectiveness of clinical trials. There has been a marked increase in basic research on brain-targeted drug delivery systems for GBM, with many innovative studies showing promising results in animal models. However, clinical translational research for these delivery systems remains limited. Increasing the focus on the progress of clinical trials for brain-targeted drug delivery systems in GBM treatment could significantly advance effective therapies for GBM. The accompanying table (Table 4) provides a comprehensive overview of the progress in pivotal trials of brain-targeted delivery systems for GBM treatment, indicating that most trials are still in the Phase I/II stages. Although research on drug delivery carriers for GBM began 20 years ago, early studies primarily focused on using liposomes to reduce drug toxicity and improve pharmacokinetic characteristics. Subsequently, attention shifted to drug carriers capable of penetrating the BBB, with EGFR receptor-mediated and LRP-1 receptor-mediated drug carriers demonstrating effectiveness in preliminary clinical trials. In recent years, the number of clinical trials has gradually increased, but there are still relatively few cases of successful translation from basic research to clinical application. Among these clinical trials, two key studies are particularly noteworthy.

**Table 4:** Comprehensive overview of clinical trials for brain drug delivery system in the treatment of GBM (2000–2024).

ClinicalTrials.Gov identifier	Methods to Overcome the BBB	Drug	Drug delivery vehicles	Pathology	Clinical Phase	Trail status	Results/Purposes	Ref.
NCT00734682	–	irinotecan	irinotecan liposome (nal-IRI)	high-grade glioma cohort was stratified into two groups based on UGT1A1 status	Phase I	Completed (2008–2015)	No unexpected toxicities were observed post intravenous administration of Nal-IRI, and there was no correlation between UGT1A1 genotype and toxicity.	[209]
NCT00944801	–	Caelyx™ (doxorubicine)	Pegylated liposomal Doxorubicine (PLD)	GBM	Phase I/II	Completed (2002–2009)	Although the combination of TMZ and PLD is well-tolerated, it does not offer a significant clinical benefit in terms of 6-month PFS and OS outcomes.	[[210], [211], [212]]
NCT03119064	–	irinotecan	nanoliposomal irinotecan	GBM	Phase I	Terminated. (2017–2021)	The dose-limiting toxicities included diarrhea and neutropenia. No significant activity was observed during the interim analysis, leading to the early termination of the study.	[213]
NCT01475006	EGFR-mediated endocytosis	DM1	antibody–drug conjugate	recurrent glioma	Phase I	Completed (2012–2016)	AMG 595 showed promising pharmacokinetic properties and holds potential benefits for certain patients with EGFRvIII-mutated GBM.	[214]
NCT03603379	EGFR-mediated endocytosis	DOX	Anti-EGFR-immunoliposomes	GBM without IDH mutation and lacking loss of heterozygosity of 1p/19q.	Phase I	Completed (2018–2020)	Anti-EGFR immunoliposomes can deliver doxorubicin specifically to EGFR-amplified GBM, potentially impacting the treatment outcome positively.	[215]
NCT02687386	EGFR-mediated endocytosis	mitoxantrone	EGFR-Erbitux EDVsMIT	recurrent glioma exhibiting EGFR expression	Phase I	Completed (2016–2020)	The utilization of EGFR-targeted minicells loaded with mitoxantrone is demonstrated to be safe and well-tolerated in pediatric patients.	[216]
none	Angiopep −2	ANG1005	Brain-penetrating peptide–drug conjugate	grades 2 to 4 glioma	Phase I	Completed (2013)	GRN1005 can deliver PTX across the BBB and achieve therapeutic concentrations in tumor tissue to effectively treat GBM.	[205]
none	Angiopep −2	ANG1005	Brain-penetrating peptide–drug conjugate	solid tumors with brain metastases	Phase II	Completed (2012)	GRN1005 exhibited good tolerability and demonstrated effectiveness in advanced solid tumors, particularly in cases involving brain metastases.	[206]
NCT01967810	Angiopep −2	ANG1005	Brain-penetrating peptide–drug conjugate	recurrent high-grade glioma	Phase II	Completed (2013–2017)	ANG1005 exhibits a favorable safety profile, is well-tolerated, and shows promising activity in recurrent malignant glioma.	[203]
NCT02048059	Angiopep −2	ANG1005	Brain-penetrating peptide–drug conjugate	recurrent brain metastases from breast cancer	Phase II	Completed (2014–2017)	All patients exhibited significant responses to both CNS and systemic treatments, resulting in improved symptoms and prolonged OS.	[202]
NCT03020017	scavenger receptors (SR)-mediated endocytosis	siRNA (Bcl2L12)	Spherical Nucleic Acid (SNA)	GBM	Phase I	Completed (2017–2018)	SNAs administered intravenously successfully targeted patient tumors. The uptake of SNAs into glioma cells was associated with a decrease in Bcl2L12 protein expression linked to the tumor.	[208]
NCT04587830	–	arginine deiminase (ADI)	ADI-PEG	GBM	Phase II	Active (2020-)	The addition of ADI-PEG 20 to RT and TMZ was found to be safe, although there may be an increased risk of. anaphylaxis and vasculitis. The preliminary OS data are encouraging.	[217]
NCT04881032	–	AGuIX nanoparticles	AGuIX nanoparticles	GBM	Phase I/II	Active (2022-)	Utilizing AGuIX nanoparticles could potentially overcome the radioresistance observed in the treatment of newly diagnosed GBM.	[218]
NCT02340156	–	SGT-53	cationic liposome	recurrent GBM	Phase II	Terminated (2014–2018)	The aim of this trial is to evaluate the 6-month PFS, OS, anti-tumor activity, safety, nanoparticle delivery to GBM.	_
NCT04573140	–	RNA-LP vaccines	RNA loaded lipid particles	MGMT unmethylated GBM	Phase I/II	Recruiting (2021-)	The objective of this study is to demonstrate manufacturing feasibility and safety, as well as to determine the maximum tolerated dose.	[219]
NCT04590664	–	verteporfin	liposomal verteporfin	EGFR + GBM	Phase I/II	Recruiting (2021-)	To evaluate the efficacy of Visudyne in treating patients with recurrent high-grade GBM associated with EGFR mutations.	–
NCT05768919	–	curcumin	liposomal curcumin (LC)	high-grade glioma	Phase I/II	Recruiting (2023-)	To evaluate the tolerability, safety, and efficacy of LC in combination with RT and TMZ in patients newly diagnosed GBM.	–
NCT05864534	ultrasound emitters	DOX	liposomal DOX	GBM	Phase II	Recruiting (2024-)	The study aims to determine the safety and feasibility of administering immune-modulating drugs via ultrasound, and assess the efficacy of this treatment for GBM.	–
NCT06356883	intraarterial	carboplatin and DOX	liposomal DOX	recurrent GBM	Phase II	Recruiting (2024-)	Investigate the impacts of intra-arterial administration of carboplatin and liposomal DOX in the treatment of GBM.	–
NCT06389591	–	pp65 RNA-LPs	RNA-Lipid Particle	recurrent GBM	Phase I/II	Recruiting (2024-)	The study aims to ascertain the manufacturing feasibility, safety, and MTD of RNA-LP vaccines in adult patients with recurrent GBM.	–
NCT06572475	–	USPIO (Ultra-Small Superparamagnetic Iron Oxide)	USPIO	vestibular schwannoma, glioma	Phase I	Recruiting (2024-)	Investigate the utility of USPIO-enhanced MRI imaging in GBM.	–
NCT05660408	–	pp65 RNA-LP (DP1)	pp65 RNA LP	recurrent high-grade glioma	Phase I/II	Not yet recruiting (2024-)	Examine the manufacturing feasibility, safety, and immunologic activity of the RNA-LP vaccine in patients with recurrent high-grade glioma.	–
NCT06477939	–	trasncrocetin	liposomal Transcrocetin (L-TC)	GBM	Phase III	Not yet recruiting (2025-)	Investigate the impact of Liposomal Transcrocetin on the treatment of GBM with radiotherapy and TMZ chemotherapy.	–
NCT01985256	–	retrovirus	Toca 511 vector	recurrent GBM	Phase I	Completed (2014–2016)	To assess the safety and tolerability of Toca 511 when administered intravenously to patients with recurrent or progressive gliomas.	[220]
NCT01844661	–	Icovir-5 (oncolytic adenovirus)	MSCs	neuroblastoma	Phase I/III	Completed (2013–2016)	The utilization of MSCs could serve as a potential strategy to enhance the delivery of oncolytic viruses to patients, thereby reducing potential toxicities and obviating the need for direct tumor injections.	[221]
NCT04758533	–	Icovir-5 (oncolytic adenovirus)	MSCs	medulloblastoma, diffuse intrinsic pontine glioma	Phase I/II	Recruiting (2021-)	To evaluate the safety, tolerability, and initial efficacy of AloCELYVIR.	–
NCT03896568	tumor targeted oncolytic adenovirus	Oncolytic adenovirus DNX-2401	MSCs	recurrent GBM	Phase I	Recruiting (2019-)	To investigate the molecular and cellular mechanisms underlying the homing and delivery of allogeneic BM-hMSCs-DNX2401 via intra-arterial administration to recurrent GBM.	–

Angiochem is a biotechnology company focused on developing novel peptide-drug conjugates PDCs that can penetrate the BBB. The company is involved in the development of ANG1005, a drug comprising paclitaxel molecules covalently linked to the Angiopep-2 peptide, currently in clinical trials [[202], [203], [204], [205], [206]]. Clinical evidence suggests that ANG1005 can delivers paclitaxel across the BBB and alleviate symptoms in patients with GBM or metastatic breast cancer tumors and extend their survival, potentially becoming the first PDC to specifically deliver paclitaxel to the brain [202,205]. Another example of successful translation from basic to clinical research is the work by researchers at Northwestern University on brain-penetrating RNA interference spherical nucleic acids (SNAs). Initial studies demonstrated the safety and efficacy of SNAs [207]. Researchers then advanced to early Phase I clinical trials, evaluating pharmacology, toxicokinetics, and drug safety in cynomolgus monkeys and humans [208].

**Fig. 7:**
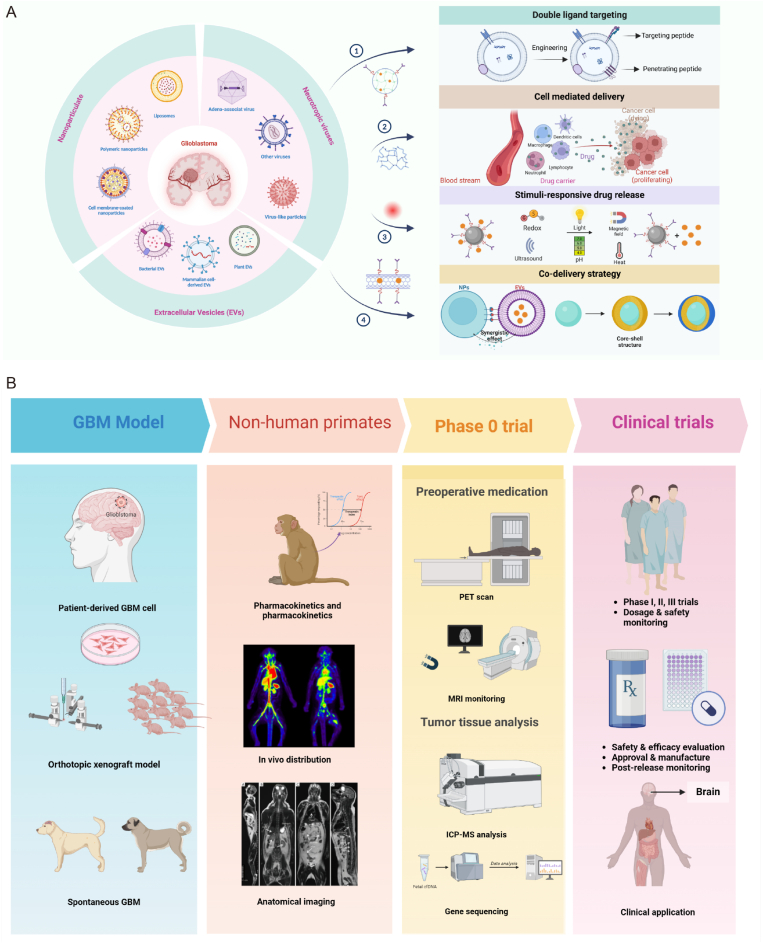
The prospects for designing drug delivery systems include advancing these systems to clinical applications for the treatment of GBM.

Based on the aforementioned research, we propose a strategy for translating basic research into clinical applications, which involves several key steps as shown in Fig. 7B. Initially, tumor models should be developed using patient-derived cell lines and expanded to include larger cohorts of mice. Employing various animal tumor models is crucial to provide robust evidence of the efficacy and safety of drug delivery systems. Following successful preclinical studies, early safety trials in non-human primates, such as cynomolgus monkeys, are necessary to ensure drug safety. Once these trials are completed, GBM patients can be ethically recruited for early clinical studies. These studies should include preoperative drug administration, with techniques such as PET or MRI employed to evaluate the in vivo distribution of drug carriers. During surgery, excised tumor tissues should be analyzed using methods like ICP-MS, fluorescence microscopy, and genetic testing to confirm the drug's targeting ability and efficacy. Finally, comprehensive large-scale clinical trials should be conducted, paving the way for the treatment's application in GBM patient therapy.

## 8 Conclusion

Brain-targeting drug delivery systems hold great promise for the treatment of GBM. These systems offer improved drug stability, targeting, and bioavailability, allowing for effective penetration of the BBB/BTB. These systems reliable platforms for diverse treatment modalities, including chemotherapy, gene therapy, immunotherapy, and combination therapies for tumor treatment. However, the primary challenge remains the low efficiency of brain targeting, which hampers precise lesion targeting within the brain. Enhancing precision in drug delivery systems necessitates the incorporation of targeting ligands into nanocarriers and the careful selection of optimal targets. Ensuring drug safety is paramount, requiring comprehensive investigations into the long-term effects of intravenously administered drug delivery systems to minimize adverse effects on normal tissues.

Although brain-targeting systems have demonstrated efficacy in inhibiting GBM growth, substantial efforts are required before clinical application. Preclinical studies primarily rely on cellular and animal tumor models, which may not accurately reflect clinical scenarios. Therefore, increased use of patient-derived tumor xenograft models is crucial for improving the relevance of anti-tumor experimentation and expediting the selection of suitable drug formulations for clinical translation.

Advancements in comprehending the structure, composition, and permeability of BBB/BTB are crucial in identifying and evaluating factors that influence the efficacy of GBM-targeted drug delivery. The mechanisms of multidrug resistance in GBM are highly intricate, potentially involving multiple physiological systems, which can make achieving optimal reversal of resistance challenging. Therefore, it is imperative to focus on the ongoing research developments in GBM treatment and their integration with effective brain-targeting drug delivery systems. As tumor treatment methods continue to advance, the management of GBM will reach unprecedented levels. Nonetheless, this journey is arduous and demanding, requiring sustained dedication and effort. Through an inductive analysis of current challenges and the use of innovative research, the review provides important guidance for the development of more effective and targeted treatment strategies for GBM, with the potential to improve patient outcomes.

## CRediT authorship contribution statement

**Bo Sun:** Writing – review & editing, Writing – original draft. **Rong Li:** Writing – review & editing, Supervision, Investigation. **Ning Ji:** Visualization, Resources, Investigation. **Han Liu:** Methodology, Investigation. **Hongxiang Wang:** Project administration, Investigation. **Chao Chen:** Supervision, Resources. **Long Bai:** Supervision, Funding acquisition. **Jiacan Su:** Supervision, Conceptualization. **Juxiang Chen:** Supervision, Investigation, Funding acquisition, Conceptualization.

## Declaration of competing interest

The authors declare that they have no known competing financial interests or personal relationships that could have appeared to influence the work reported in this paper.

## Data Availability

No data was used for the research described in the article.
